# UPF1-mediated mRNA decay promotes melanoma tumorigenesis through miR-101-3p-dependent destabilization of NANOS1

**DOI:** 10.7150/ijbs.134804

**Published:** 2026-09-24

**Authors:** Suman Lee, Jae-Won Yoon, Dayeon Woo, Hyunho Cha, Seungwon Jung, Je-Min Choi, Jungwook Hwang

**Affiliations:** 1 Graduate School for Biomedical Science & Engineering, Hanyang University, Seoul, Korea.; 2 Department of Life Science, College of Natural Sciences, Hanyang University, Seoul, Korea.; 3 Hanyang Institute of Bioscience and Biotechnology, Hanyang University, Seoul, Korea.; 4 Research Institute for Natural Sciences, Hanyang University, Seoul, Republic of Korea.; 5 Research Institute for Convergence of Basic Sciences, Hanyang University, Seoul, Republic of Korea.

**Keywords:** melanoma, UPF1, NANOS1, miR-101-3p, ELK1, drug resistance

## Abstract

Numerous studies have reported Up-frameshift protein 1 (UPF1) as a crucial posttranscriptional regulator in nonsense-mediated mRNA decay (NMD), attributing its role to tumorigenesis across various cancers. However, its recently unveiled function in UPF1-mediated mRNA decay (UMD), intricately coordinated with miRNA, remains unexplored in the context of tumorigenesis. Here, we demonstrate that UPF1 contributes to melanoma tumorigenesis by regulating NANOS1 expression in a miR-101-3p-dependent manner. Depletion of UPF1 across three melanoma cell lines significantly impaired cell growth, migration, and invasion by cell cycle arrest and disrupted tumor formation *in vivo*. Through comprehensive RNA-seq and biochemical analyses, we elucidate that attenuation of UPF1 leads to an increase in NANOS1 expression, consequently suppressing melanoma tumorigenesis. Mechanistically, UPF1 orchestrates the destabilization of NANOS1 via UPF1-mediated mRNA decay (UMD) facilitated by miR-101-3p. Intriguingly, UPF1 depletion and NANOS1 upregulation were associated with reduced ERK and ELK1 phosphorylation, suggesting that attenuation of MAPK/ERK/ELK1 signaling may contribute, at least in part, to the observed melanoma-suppressive phenotypes. Importantly, the biological relevance of UPF1 and NANOS1 in melanoma cell lines was further supported in drug-resistant melanoma cells and in xenograft mouse models. Collectively, these findings expand our understanding of UMD in melanoma and identify the UPF1/NANOS1 regulatory relationship as a potential therapeutic vulnerability in both parental and drug-resistant melanoma cells.

## Introduction

Melanoma stands out as one of the most formidable cancers, and cutaneous melanoma in particular accounts for the majority of skin cancer-related fatalities 1, 2. While various factors contribute to the development of melanoma, genetic alterations induced by UV exposure have emerged as predominant instigators 3. Extensive genetic analyses have underscored the BRAF and NRAS mutations along with NF1 deficiency as major contributors to melanoma, which exhibits metastatic potential to populate diverse anatomical sites, including the adrenal glands, bone, brain, gastrointestinal tract, heart, liver, lung, and lymph nodes 4-12. Targeting the mitogen-activated protein kinase (MAPK) pathway, BRAF and MEK inhibitors, such as vemurafenib and trametinib, have significantly improved overall survival in melanoma patients 13-15. However, the development of drug resistance remains a major challenge in their long-term efficacy 15, 16. The exploration of alternative therapeutic strategies and mechanisms underlying MAPK inhibitor resistance has gained momentum to address this issue 17-19. Recently, immune checkpoint inhibitors, including cytotoxic T lymphocyte antigen 4 (CTLA4) and programmed death-1 (PD-1), have been approved for melanoma treatment, demonstrating improved survival rates and durable responses in patients 20.

Many studies have revealed that nonsense-mediated mRNA decay (NMD) has a function in tumorigenesis in multiple tumors 21-23. NMD is a post-transcriptional regulatory mechanism with expected primary function for quality control of mutant transcripts with premature termination codons (PTCs) in an exon junction complex (EJC)-dependent manner. NMD has also recently been found to regulate expression of wild-type transcripts that lack PTCs and EJCs 24-28. Although removal of long 3'UTR-containing or EJC-null transcript by NMD is not fully understood, recent studies suggest its association with conserved microRNA (miRNA) recognition elements (MREs) and their regulation by binding miRNAs. This regulation occurs through formation of a complex between up-frameshift protein 1 (UPF1) and Argonaute2 (AGO2) and miRNA, named UPF1-mediated mRNA decay (UMD) 29, 30. Furthermore, UPF1 exhibits aberrant expression in various cancers, including hepatocellular carcinoma, colorectal cancer, pancreatic carcinoma, ovarian cancer, and glioblastoma 31-37. Despite the implication of UPF1 in diverse cancers, the role of UMD in cancers remains elusive. Another RNA binding protein, NANOS1, a single-exon gene encoding a C2HC-Type zinc finger protein with a long 3'UTR, is recognized for its role in regulating transcripts containing the Pumilio Regulatory Motif (PRE) in the 3'UTR and regulating translation as a post-transcriptional repressor 38-41. Because NANOS1 has been studied primarily in developmental biology but rarely in cancer biology, its role in melanoma remains unknown 42-44. ELK1, a well-known transcription factor, is a member of the E twenty-six (ETS) oncogene family that is activated by ERK, p38, and Jun 45-47. In addition, the phosphorylation of ELK1 is an important link in the MAPK signaling which is involved in cell growth, differentiation, survival, inflammation and tumorigenesis 48, 49.

Our study identifies UPF1 and NANOS1 as novel candidate genes for malignant melanoma treatment. We demonstrate that the downregulation of UPF1 hampers malignant melanoma proliferation through posttranscriptional regulation of NANOS1. UPF1 governs NANOS1 expression via miRNA binding to the NANOS1 3'UTR. Expression of NANOS1 impedes melanoma proliferation by inducing cell cycle arrest. Significantly, depleted UPF1 and elevated NANOS1 levels exhibit inhibitory effects on the tumorigenesis of drug-resistant cells. Furthermore, depletion and induction of UPF1 and NANOS1, respectively, reduce tumor formation and metastasis in *in vivo* models. The inhibitory effects of UPF1 depletion and NANOS1 upregulation on melanoma tumorigenesis were associated with reduced ERK and ELK1 phosphorylation, potentially through the association of NANOS1 with MAPK signaling. Thus, our findings propose UPF1 and NANOS1 as promising and innovative targets for drug-resistant melanoma gene therapy.

## Materials and Methods

### Cell culture, transfection, and infection

Three melanoma cell lines (A375, SK-MEL-2, and SK-MEL-28, all purchased from the Korean Cell Line Bank) and MEL-ST cell lines (kindly gifted by Dr. Weinberg at MIT) 50 were maintained in Dulbecco's modified Eagle's medium (DMEM) supplemented with 10% fetal bovine serum and 1% penicillin/streptomycin. Primary epidermal melanocytes were purchased from ATCC (PCS-200-013, VA, USA) and maintained according to the manufacturer's protocol. To deplete or overexpress the target gene, cells were transfected with the indicated siRNA or DNA plasmid using Lipofectamine 3000 (Invitrogen, MA, USA). Control siRNA or an empty vector was transfected as a negative control. Unless otherwise indicated, cells were harvested 48 h after siRNA or DNA plasmid transfection for RT-qPCR and western blot analysis. Cell growth assays were initiated 24 h after transfection, and cell proliferation was monitored at the indicated time points using the Cell Counting Kit-8 assay (CCK8, Dojindo, Japan) or direct cell counting, as indicated in the corresponding figure legends. The specific siRNA sequences are listed in Table S2. For rescue experiments, cells were co-transfected with the indicated siRNA and plasmid constructs. For retrovirus production, the EGFP-IRES-C5W2 vector containing the NANOS1 was transfected into 293GPG cells, and virus-containing culture supernatants were collected for 7 days. For lentivirus production, the pGIPz vector containing the human or mouse shUPF1 and the pCDH vector containing the NANOS1 were co-transfected with lentiviral packaging plasmids psPAX2 and pMD2.G into 293T cells. Virus-containing supernatants were collected 72 h after transfection, concentrated using PEG buffer, and used to infect target cells in the presence of polybrene. Stable knockdown cells were selected using puromycin (1 ug/ml). To determine posttranscriptional regulation, transcription was inhibited with 100 μg/mL 5,6-dichloro-1-β-D-ribofuranosylbenzimidazole (DRB), and cells were harvested at the indicated time points after DRB treatment for RT-qPCR.

### Generation of drug-resistant melanoma cells

Drug-resistant melanoma cells were generated by stepwise selection under increasing concentrations of the relevant inhibitors. A375 and SK-MEL-28 cells were used to establish BRAFi-resistant lines by initial exposure to 0.5 μM vemurafenib (Cell Signaling, 17531, MA, USA), followed by dose escalation of 0.2 μM every 3 days to a final concentration of 5 μM. SK-MEL-2 cells were used to generate MEK inhibitor-resistant (MEKi-resistant) lines by initial treatment with 0.5 nM trametinib (Cell Signaling, 62206, MA, USA), followed by dose escalation of 0.1 nM every 3 days to a final concentration of 2 nM. Resistant cells were thereafter continuously maintained in medium supplemented with the corresponding final inhibitor concentration.

### Plasmid construction

To construct C-terminally FLAG-tagged NANOS1, the coding sequence (CDS) was synthesized by annealing serial oligonucleotide methods, which contained HindIII and BamHI sites at 5' and 3' ends, respectively. NANOS1 CDS was digested with HindIII and BamHI and ligated to the digested pcDNA3.1 vector fragment with the same digestion enzymes (pcDNA3.1-NANOS1). Then, oligo-synthesized FLAG containing BamHI and EcoRI at 5' and 3' ends was digested with BamHI and EcoRI and ligated to digested pcDNA3.1-NANOS1 vector with BamHI and EcoRI (pcDNA3.1-NANOS1-FLAG).

To construct the EGFP vector with various lengths of NANOS1 3'UTR, EGFP was amplified with EGFP-F and EGFP-R primer containing HindIII and BamHI sites at the 5' and 3' ends, respectively, and ligated into the pcDNA3.1vectors digested with HindIII and BamHI. Then various lengths of NANOS1 3'UTR PCR product, amplified from A375 genomic DNA and two pairs of primers, where -1, -2, and -3 represented the length of 3'UTR (Figure 6A), were ligated into a pcDNA3.1-EGFP vector digested with BamHI and XhoI.

To construct the human NANOS1 3'UTR reporter vector, synthesized NANOS1-3'UTR-WT and NANOS1-3'UTR-Mut oligo were amplified using NANOS1-3'UTR-miRNA-F and NANOS1-3'UTR-miRNA-R primer and inserted by In-Fusion into the pmirGLO vector digested with XbaI.

To construct the pFLAG-ELK1 WT vector, the ELK1 coding sequence (CDS) was amplified from A375 cells cDNA using ELK1-F and ELK1-R primers and inserted by In-Fusion into a pFLAG vector digested with HindIII. Then, site-directed mutagenesis methods were employed with ELK1-Mut-F and ELK1-Mut-R primers to construct the pFLAG-ELK1 383 (S>A) mutation vector.

To construct the human and mouse UPF1 knock-down lentivirus vectors, synthesized human and mouse shRNA oligos were inserted into a pGIPz vector digested with XhoI by In-Fusion methods.

To construct the human NANOS1 overexpression lentivirus vector, the hygromycin (Hygro)-resistant sequence was amplified from the pCF525 vector using Hygro-F and Hygro-R primer and inserted by In-Fusion into the pCDH vector digested with EcoRI and SalI. Then, the NANOS1-FLAG sequence was amplified from pcDNA3.1-NANOS1 vector using NANOS1-Lenti F and NANOS1-Lenti R primer and inserted by In-Fusion into the pCDH-Hygro vector digested with SalI.

To construct the human NANOS1 overexpression retrovirus vector, the NANOS1-FLAG sequence was amplified from pcDNA3.1-NANOS1 vector using NANOS1-Retro F and NANOS1-Retro R primers and inserted by In-Fusion into the EGFP-IRES-C5W2 vector digested with XhoI. All primers and oligos are listed in Table S3 and Table S4.

### Western blotting (WB) analysis

To detect specific proteins, proteins in total cell lysates were eluted with SDS and β-mercaptoethanol. Then, the proteins were separated on gels containing various percentages of polyacrylamide and transferred to nitrocellulose membranes. Blotting was performed with antibodies specific for the following proteins: UPF1 (Cell Signaling, 12040, MA, USA), NANOS1 (MyBioSource, MBS648789, CA, USA), Calnexin (Cell Signaling, 2679, MA, USA), EGFP (Santa Cruz, sc-9996, TX, USA), FLAG (Sigma, F3165, MO, USA), Caspase-3 (Cell Signaling, 9662, MA, USA), Cleaved-Caspase-3 (Cell Signaling, 9661, MA, USA), ERK (Cell Signaling, 9102, MA, USA), p-ERK (Cell signaling, 9101, MA, USA), ELK1 (Cell signaling, 51398, MA, USA), p-ELK1 (Cell signaling, 9181, MA, USA), p-PDK1 (Cell Signaling, 3061, MA, USA), and β-actin (Sigma, A2228, MO, USA).

### RT-qPCR

Total RNA was extracted using TRIzol (Invitrogen, MA, USA). To remove exogenous and endogenous DNA, extracted RNA was treated with RQ DNase I (Promega, WI, USA). cDNA was synthesized with RTase (ThermoFisher, MA, USA) using random hexamer primers (Macrogen, Korea). For miRNA reverse transcription, the specific stem-loop RT primer (Macrogen, Korea) was used. RT-qPCR was performed using the primers listed in Table S5 and Table S6.

### Immunohistochemical staining

Malignant melanoma tissue array slide was purchased from tissuearray.com (T383a, MD, USA). Paraffin-embedded tissue sections were deparaffinized in xylene and rehydrated through a graded ethanol series. Antigen retrieval was performed by heating the sections in citrate buffer (10 mM, pH 6.0) using a microwave oven for 15 minutes. Endogenous peroxidase activity was blocked by incubating the sections with 3% hydrogen peroxide in PBS for 5 minutes at room temperature. After washing with PBS, non-specific binding was blocked by incubating the sections with 5% normal goat serum for 20 minutes at room temperature. The sections were then incubated 1 hour at room temperature with the primary antibody, followed by washing with PBS and incubation with a biotinylated secondary antibody (65-6140, Invitrogen, MA, USA) for 20 minutes at room temperature. This was followed by incubation with streptavidin-horseradish peroxidase conjugate (Sigma, OR03L, MO, USA). The antigen-antibody complexes were visualized using a DAB (3,3'-diaminobenzidine tetrahydrochloride hydrate) (Sigma, D5637, MO, USA) according to the manufacturer's instructions. Hematoxylin was used as a counterstain. Sections were dehydrated, cleared, and mounted with a coverslip. Images were captured using a digital slide scanner (Carl Zeiss, Axioscan 7, Germany).

### Migration and invasion assay

To evaluate cell migration, melanoma cells transfected with the indicated siRNA or plasmid were cultured for 48 h after transfection. Confluent cell monolayers were then scratched with a sterile pipette tip, and wound closure was assessed 24 h after scratching. The migrated area was quantified using AxioVision Rel 4.8 software. For invasion assays, cells were harvested 48 h after transfection, resuspended in serum-free medium, and seeded into invasion chamber inserts containing Matrigel-coated membranes (Corning, NY, USA). After 24 h of incubation, cells that had invaded through the membrane were fixed, visualized by hematoxylin and eosin staining, and quantified.

### Immunofluorescence

Cells seeded on coverslips were fixed with 3.7% formaldehyde and permeabilized with PBS containing 0.2% saponin. After three washes and blocking with 3% BSA, cells were incubated with primary antibodies, followed by secondary antibodies (Alexa488, Life Technologies). Nuclei were visualized by staining with DAPI. Cells were stained with the specific PCNA (Santa Cruz, 9129, TX, USA) and Ki67 (Cell Signaling, SC-56, 9129, MA, USA) antibodies.

### Flow cytometry

To evaluate the cell cycle in UPF1-depleted or NANOS1-FLAG-expressing A375 cells, a 5-bromo-2'-deoxyuridine flow kit (BD Pharmingen, CA, USA) was employed. Three days after transduction, cells were incubated with 10 μM BrdU prior to fixation. Then, 7-aminoactinomycinD (7-AAD) was added. Cell cycle phases were determined using flow cytometry (FACSCanto, BD Pharmingen, CA, USA). To analyze the apoptotic cell death, an Annexin V-FITC Apoptosis Detection Kit (556547, BD Pharmingen, CA, USA) was employed. Briefly, a total of 100 μL of the cell suspension was transferred to a 5 mL flow cytometry tube, followed by the addition of 5 μL Annexin V-FITC and 5 μL propidium iodide (PI), and incubated for 15 minutes at room temperature in the dark. After incubation, 400 μL of 1× binding buffer was added to each tube. Stained cells were analyzed using a flow cytometry (FACSCanto, BD Pharmingen, CA, USA).

### Xenograft model

For the xenograft model, 7-week-old male BALB/c nude (CAnN.Cg-Foxn1 nu/Crl) mice were purchased from Charles River Laboratories. To generate stable cell lines that were UPF1-depleted or NANOS1-FLAG-overexpressing, shUPF1-expressing lentivirus or NANOS1-expressing retrovirus was infected into A375 cells for 3 days. Lentivirus-infected cells were selected using puromycin (1 μg/ml) for 3 days. Then, 2 × 10^6^ of the selected cells were subcutaneously injected into each left and right sides of the mouse flanks. Tumor growth was tracked weekly by caliper measurement, and tumor volume was calculated using [volume] = 0.52 x [length] x [width]^2^. Mice were euthanized when control tumors reached approximately 1000mm^3^, and tumors were harvested for further experiments. Mice were housed and bred in a specific pathogen-free animal facility at Hanyang University under controlled conditions with a constant temperature (21 ± 1 °C) and humidity (50 ± 5%) and a 12-h light/dark cycle with regular chow and autoclaved water. All mouse experimental procedures used in this study were approved by the Institutional Animal Care and Use Committee of Hanyang University (2023-0132A, 2024-0004A). Studies of human cell line in xenograft model were exempted by the Institutional Review Board of Hanyang University (HYU-2024-152).

### Pulmonary melanoma metastasis model

The B16F10 mouse melanoma cell line, derived from C57BL6/J mouse melanoma, was acquired from ATCC (CRL-6475, VA, USA). B16F10 cells were infected with shUPF1-expressing lentivirus or human NANOS1-expressing lentivirus for 3 days, followed by selection with puromycin (5 μg/ml) or hygromycin (600 μg/ml) for 3 days. After selection, cells were harvested, and 5 x 10^5^ cells were intravenously injected into age- and sex-matched 8- to 10-week-old C57BL6/J mice. On day 14, mice were euthanized, and the number of melanoma colonies visualized as black dots on the lung surface was counted.

### Statistical analysis

For simple comparisons between two independent groups, two-sided unpaired Student's t-tests were used as appropriate. Data are presented as mean ± SD from independent experiments. Cell growth curves, including rescue assays, were analyzed using two-way ANOVA with treatment and time as factors, followed by Šídák's multiple-comparison test for comparisons between groups at each time point. Tumor growth curves measured over time were analyzed using a repeated-measures or mixed-effects model, followed by Šídák's multiple-comparison test where appropriate. For cell-cycle analyses, treatment-associated changes were evaluated based on the overall cell-cycle phase distribution, and P-values were adjusted using Bonferroni correction where statistical comparisons were presented. P-values for volcano plots were obtained using the local-pooled-error (LPE) test. Statistical significance is indicated in the figures as follows: *P ≤ 0.05; **P ≤ 0.01; ***P ≤ 0.001; ns, not significant.

### RNA-seq and data analyses

Total RNA from the cell lysates that were transiently transfected with control siRNA or siUPF1 was isolated using Trizol following the manufacturer's instruction. To assess the integrity of the total RNA, samples are run on the TapeStation RNA screentape (Agilent Technologies, CA, USA) at the Biospecimen-Multiomics Digital Bioanalysis Core Facility of Hanyang University. A library was independently prepared with 1 μg of total RNA for each sample by Illumina TruSeq Stranded mRNA Sample Prep Kit (Illumina, Inc., CA, USA). The libraries were quantified using KAPA Library Quantification Kits for Illumina Sequencing platforms according to the qPCR quantification protocol guide (KAPA Biosystems, MA, USA) and qualified using a TapeStation D1000 ScreenTape chip (Agilent Technologies, CA, USA). The indexed libraries were then subjected to paired-end (2x100bp) sequencing on the Illumina NovaSeq platform (Illumina, Inc., CA, USA) by Macrogen Incorporated. The raw reads after removal of low-quality and adapter sequences were aligned to the Homo sapiens sequence (GRCh 37) using HISAT2. Based on the mapping results, the numbers of transcripts and genes were calculated as the read counts or fragments per kilobase of exon per million fragments mapped (FPKM) values for each sample by StringTie. The expression profiles were used to perform additional analyses, such as differentially expressed genes (DEGs) and Gene Ontology (GO) analysis. Our RNA-seq transcriptome data have been deposited in NCBI BioProjects GSE267965.

### RIP-seq and data analyses

A375 melanoma cells were transfected with either FLAG control plasmid or NANOS1-FLAG expression plasmid. At 48 h after transfection, cells were harvested. Ten percent of each lysate was saved as the input sample, and the remaining lysates were incubated with anti-FLAG antibody-conjugated beads (Sigma, A2220, MO, USA) at 4°C with gentle rotation. After immunoprecipitation, the beads were washed to remove nonspecifically bound materials, and RNA was isolated from both input and immunoprecipitated fractions. Purified RNA was used for RNA-seq library preparation using the NEBNext Ultra II Directional RNA Library Prep Kit (NEB, #E7760L, MA, USA) according to the manufacturer's instructions. Libraries were sequenced on an Illumina platform. A total of 12 libraries were generated and sequenced: FLAG input samples (n = 2), NANOS1-FLAG input samples (n = 2), FLAG RIP samples (n = 4), and NANOS1-FLAG RIP samples (n = 4). Sequencing reads were aligned to the human reference genome (GRCh38) using STAR, and gene-level expression estimates were obtained using RSEM. To identify transcripts enriched in NANOS1-FLAG RIP samples, RSEM count matrices were analyzed using DESeq2. Differential RIP enrichment was assessed using an interaction model comparing IP/input enrichment between NANOS1-FLAG and FLAG control samples. Input-only differential expression analysis was also performed by comparing NANOS1-FLAG input samples with FLAG input samples using DESeq2 to determine whether NANOS1-FLAG overexpression altered baseline transcript abundance.

### Long-read sequencing

The Oxford Nanopore Technology (ONT, UK) SQK-DCS109 kit was used for direct cDNA sequencing as described in the manufacturer's instructions. Briefly, 750 ng of poly(A) containing mRNA was used as input. The libraries were sequenced on a MinION using R9.4.1 flowcells and with the MinKNOW instrument software. Fast5 files were generated, and basecalling of the fast5 files was performed using GUPPY version (1.4.3-1 from ONT, UK).

### Phospho Explorer antibody microarray

The Phospho Explorer antibody array assay was performed by Full Moon Biosystems on UPF1-depleted A375 cells according to the manufacturer's protocol. Briefly, cell lysates that were prepared using a Protein Extraction Kit (Full Moon Biosystems, CA, USA) were biotinylated and incubated with Phospho Explorer Antibody Arrays, which contain 1,318 antibodies. The slides were then incubated with Cy3-streptavidin, and the array was scanned on a GenePix 4100A Array Scanner (Molecular Devices, CA, USA). Image quantification was performed on GenePix 7.0 (Molecular Devices, CA, USA). Each antibody had two replicates, and the mean signal intensity of the replicates was determined. The data were normalized to the median value of all antibodies, and the fold change between the control and treatment samples was determined using the normalized data.

## Results

**UPF1 regulates melanoma tumorigenesis *in vitro* and *in vivo*.** Given UPF1's role in various cancers, we first analyzed the Oncolnc database to assess tumor patient survival rates, revealing that low *UPF1* expression in Skin Cutaneous Melanoma (SKCM) patients was associated with improved overall survival (Figure S1A). Consistent with this observation, TCGA SKCM analysis showed a tendency toward increased *UPF1* expression in melanoma samples compared with normal samples, although statistical significance was limited by the small number of normal samples (Figure S1B). Similarly, immunohistochemical analysis showed little to no detectable UPF1 staining in normal tissue, whereas melanoma tissues exhibited increased UPF1-positive staining (Figure S1C). Additionally, observing disrupted keratinocyte homeostasis upon UPF1 depletion 51, we hypothesized that UPF1 regulates melanoma tumorigenesis. To determine the effects of UPF1 on melanoma cells, we employed three melanoma cell lines: A375 (BRAF mutation), SK-MEL-2 (NRAS mutation), and SK-MEL-28 (BRAF mutation). UPF1 was downregulated by siRNA in the three cell lines, and cell growth was monitored for 72 hr, indicating that depletion of UPF1 in melanoma cell lines reduced cell growth (Figure 1A). Consistent with the A375 cells, UPF1 depletion also reduced the cell growth of A375SM cells, which exhibit enhanced metastatic potential (Figure S2A). These findings were confirmed by restoring UPF1 expression, upon which depleted UPF1 was recovered by siRNA-resistant UPF1-expressing-DNA plasmid, demonstrating that restoration of UPF1 alleviated retarded cell growth by UPF1-depletion (Figure 1B, Figure S2B). Given the effects of UPF1 on the growth of melanoma cells, we next determined its effects on metastatic potential and migration (Figure 1C, D). Consistent with the cell growth results, UPF1 depletion reduced invasion and migration across all three melanoma cell lines, with the magnitude of the effect varying according to the cell line and assay type. Furthermore, the depletion of UPF1 arrested the cell cycle in the G1 phase but did not cause apoptosis (Figure 1E, Figure S2C, D). Thus, we concluded that UPF1 regulates melanoma tumorigenesis.

To examine the *in vitro* observation using melanoma cell lines in a mouse model, A375 cells infected with lentiviral vectors expressing shUPF1 were injected into nude mice, and tumor volumes were monitored weekly for 6 weeks. Consistent with the *in vitro* results, depletion of UPF1 showed a decreasing pattern in tumor volume and weight in xenografted mice (Figure 2A-C, Figure S2E). Consistently, endpoint analysis of harvested xenograft tumors showed sustained UPF1 knockdown in shUPF1-derived tumors compared with control tumors (Figure S2F). Furthermore, depletion of UPF1 efficiently decreased expression of Ki-67 and PCNA, well-known proliferation markers, in xenografted tumors (Figure 2D). Then, we employed the B16F10 melanoma lung metastasis model to confirm the effects of Upf1 depletion on melanoma metastasis 52. The depletion of mouse Upf1 in the mouse melanoma cell line, B16F10, retarded cell growth (Figure S2G). Upf1-depleted B16F10 mouse melanoma cells were intravenously injected into C57BL6/J mice, and metastatic melanoma colonies were observed on the lung surface, demonstrating that melanoma lung metastasis was significantly reduced by Upf1 depletion (Figure 2E). However, in MEL-ST cells, an immortalized melanocyte cell, overexpression of UPF1 did not affect cell growth, suggesting that UPF1 has the effects on melanoma growth (Figure S2H). All these results indicate that UPF1 regulates melanoma tumorigenesis *in vitro* and *in vivo*.

**UPF1 regulates the abundance of *NANOS1* mRNA.** UPF1 is a well-known posttranscriptional regulator that causes degradation of its targets through NMD or UMD. Thus, we sought to analyze the transcriptomic changes in melanoma cells upon UPF1 depletion by RNA-seq (Figure 3A, Table S1). We identified NANOS1 as a downstream gene potentially regulated by UPF1, as transcriptome analysis consistently showed significant upregulation of *NANOS1* across all three datasets (Figure S3A). Furthermore, SKCM patients with high *NANOS1* expression exhibited higher survival rates and TCGA SKCM analysis showed that *NANOS1* expression tended to be lower in melanoma samples than in normal samples (Figure 3B, Figure S3B). To verify the upregulation of NANOS1 upon UPF1 depletion, we performed RT-qPCR and WB using UPF1-depleted melanoma cells and xenografted tumors (Figure 3C, Figure S3C), demonstrating that downregulation of UPF1 upregulated the abundance of *NANOS1* mRNA up to 3-fold. To confirm that *NANOS1* mRNA is posttranscriptionally regulated by UPF1, we measured the stability of *NANOS1* mRNA in the presence or absence of UPF1 under transcriptional inhibition with 5,6-dichloro-1-beta-D-ribofuranosylbenzimidazole (DRB). RT-qPCR results of DRB treatment indicated that depletion of UPF1 increased the half-life of *NANOS1* mRNA by approximately 4-fold but not in control housekeeping transcripts, suggesting that the abundance of *NANOS1* mRNA was posttranscriptionally regulated by UPF1 (Figure 3D, Figure S3D). Then, we compared the expression levels of UPF1 and NANOS1 using normal melanocytes and the three melanoma cell lines, results of which indicated that each melanoma cell line contains more UPF1 and less NANOS1 than melanocytes (Figure 3E). Notably, the opposite expression of *UPF1* and *NANOS1* was observed in public single-cell RNA-seq analysis using patient-derived melanoma (Figure 3F). The simple interpretation of these results suggests that UPF1 posttranscriptionally regulates the abundance of NANOS1 *in vitro* and *in vivo*.

**NANOS1 regulates melanoma tumorigenesis.** Upregulation of NANOS1 expression upon UPF1 depletion led us to address the effects of NANOS1 on melanoma tumorigenesis. We postulated that increased NANOS1 induced by UPF1 depletion downregulated melanoma tumorigenesis. First, we elucidated growth repression of melanoma cells by overexpression of C-terminally FLAG-tagged NANOS1 (NANOS1-FLAG) (Figure 4A). In support, depletion of NANOS1 in melanoma cells enhanced cell growth (Figure 4B). Next, retardation of cell growth by UPF1 depletion was canceled by NANOS1 depletion, suggesting that melanoma cell growth was modulated by UPF1/NANOS1 axis (Figure 4C). All these effects of NANOS1 on cell proliferation were recapitulated by staining a proliferation marker, Ki-67, in the presence or absence of UPF1 and NANOS1 (Figure S3E, F). In addition, all cell growth results were verified by an invasion and migration assay using the three melanoma cell lines that were transiently transfected with NANOS1-FLAG or FLAG. NANOS1 overexpression effectively reduced metastatic potential and cell migration in the three melanoma cell lines, as did the depletion of UPF1 (Figure 4D, E). Additionally, retardation of cell growth canceled by NANOS1 depletion was observed in migration assay (Figure 4F, Figure S3G). Similarly, NANOS1 overexpression also arrested the cell cycle in the G1 phase not in apoptosis (Figure 4G, Figure S3H, I). All observations indicate that NANOS1 regulates melanoma tumorigenesis. These results were verified by *in vivo* approaches. A375 cells infected with a retroviral vector expressing NANOS1-FLAG were injected into nude mice, and tumor volumes were monitored for 7 weeks. Consistent with UPF1 depletion, overexpression of NANOS1 also reduced tumor volume and weight, accompanied by decreased levels of proliferation markers Ki-67 and PCNA (Figure 5A-D, Figure S4A, B). Consistent with the metastatic results using depleted Upf1 in B16F10, the overexpression of NANOS1-FLAG also effectively abrogated melanoma lung metastasis *in vivo* (Figure 5E). Taking these findings into account, we concluded that UPF1-mediated NANOS1 regulation has effects on melanoma tumorigenesis *in vitro* and *in vivo*.

**UPF1 regulates NANOS1 expression via miR-101-3p.** UPF1 is a well-known posttranscriptional regulator that targets i) PTC-bearing transcripts by forming a complex in exon-junction complex (EJC) (EJC-dependent NMD) 53, 54 or ii) long 3'UTR-containing transcript by combination of AGO2 and SMG7 in UPF1-mediated mRNA decay (UMD) 29. Because NANOS1 is composed of a single exon, we excluded the possibility of EJC-dependent NMD and hypothesized that NANOS1 could be regulated by UMD using 3'UTR of *NANOS1*. Because human *NANOS1* mRNA is annotated to have a 3'UTR length of either 850 nucleotides (nt) or 3,050 nt, we determined the length of *NANOS1* 3'UTR in melanoma cells by short-read and long-read RNA sequencing, which indicated that 850 nt-containing 3'UTR of *NANOS1* was the major form in A375 cells (Figure S4C). Then, we generated a GFP reporter construct that contains different lengths of 3'UTR from *NANOS1* to determine which region is responsible for UPF1-mediated regulation (Figure 6A). A375 cells that were transiently transfected with GFP reporter constructs containing various lengths of 3'UTR in the presence or absence of UPF1 were employed for WB and RT-qPCR, demonstrating that depletion of UPF1 upregulated all reporter expression (Figure 6B). Because upregulation of transcripts of length between 1-850 nt, 1-1,870 nt, and 1-3,050 nt was comparable, we concluded that UPF1 regulated *NANOS1* abundance through 1-850 nt of *NANOS1* 3'UTR. In UMD, UPF1 regulates mRNA abundance with the help of SMG7 and miRNA, which contain a CAG sequence 29. To elucidate whether UPF1 regulates the level of *NANOS1* mRNA via UMD, the levels of *NANOS1* mRNA in the absence of UPF1 or SMG7 were measured by RT-qPCR. The results suggest that *NANOS1* expression was also modulated by SMG7 (Figure 6C). Then, we searched the possible miRNAs harboring 5'-CAG-3' in the seed sites that target *NANOS1* mRNA through TargetScan (www.targetscan.org). Among two putative miRNAs, miR-101-3p and miR-144-3p, we selected miR-101-3p due to its higher expression in skin than miR-144-3p (Figure 6D, Figure S4D). Moreover, the binding sites of miR-101-3p were conserved in mammals (Figure S4E). To determine whether miR-101-3p reduced *NANOS1* mRNA expression, we transiently transfected miR-101-3p mimics and compared the levels of *NANOS1* mRNA with the values in control miRNA-transfected cells by RT-qPCR, indicating that miR-101-3p reduced *NANOS1* and *HDAC9*, a well-known miR-101-3p target 55, up to 2-fold (Figure 6E). The decrease of *NANOS1* mRNA by miR-101-3p was reversed by depletion of UPF1, suggesting that miR-101-3p might regulate *NANOS1* abundance via UMD without affecting UPF1 expression (Figure 6F). Furthermore, we verified miR-101-3p levels in UPF1-depleted melanoma cells and found that UPF1 depletion did not alter miR-101-3p levels (Figure S4F). To examine the interaction between NANOS1 and miR-101-3p via AGO2, FLAG-AGO2 IP was performed. The introduction of miR-101-3p in A375 cells increased the enrichment of *NANOS1* mRNA in AGO2 IP eluates compared to the control miRNA mimic (Figure 6G). In a consistent manner, the introduction of miR-101-3p inhibitor reduced AGO2-bound *NANOS1* mRNA. In addition, the results of the RT-qPCR analysis revealed that the number of molecules of *NANOS1* per cell was approximately 315.56 molecules/cell in A375 cells, suggesting that the endogenous *NANOS1* mRNA expression was enough to regulate the fate of melanoma cells (Figure S4G). To ensure that *NANOS1* was a miR-101-3p target, we generated bicistronic reporter constructs with either wild-type (WT) or mutated (Mut) miR-101-3p binding sites in the 3'UTR of *NANOS1* (Figure 6H). The exogenous miR-101-3p mimic reduced the expression of WT miR-101-3p binding sites containing NANOS1-3'UTR but had no effect on mutant binding sites (Figure 6I). Consistent with this, the introduction of the miR-101-3p inhibitor upregulated the abundance of the WT miR-101-3p binding site-containing NANOS1-3'UTR. All of these observations were confirmed by MYC-UPF1 IP in the presence of WT or Mut reporter, indicating that MYC-UPF1 has the preference to bind more WT miR-101-3p binding than Mut (Figure 6J). Then, we examined whether miR-101-3p functionally contributes to melanoma proliferation by performing growth and migration assays in the presence of a miR-101-3p mimic or inhibitor. miR-101-3p mimic increased cell growth and migration, whereas inhibition of miR-101-3p reduced cell growth and migration. These findings support the functional relevance of miR-101-3p in melanoma growth regulation and are consistent with its role as an upstream negative regulator of NANOS1 expression (Figure S4H, I). Taken together, all results from the biochemical assays indicate that *NANOS1* is a direct target of miR-101-3p, and that *NANOS1* expression is regulated via the UMD pathway.

**The UPF1/NANOS1 axis is associated with reduced ERK/ELK1 signaling in melanoma cells.** As a first step toward determining how UMD/NANOS1 regulates melanoma tumorigenesis, we postulated that the UMD/NANOS1 axis may modulate melanoma tumorigenesis via protein modification because both UPF1 and NANOS1 were involved in MAPK signaling 56, 57. This idea was tested using an antibody microarray that could detect the phosphorylation status of cellular proteins. Intriguingly, downregulated UPF1 dramatically reduced the levels of phosphorylation of various proteins involved in MAPK signaling, most notably those of ELK1 (p-ELK1) and PDK1 (p-PDK1) (Figure 7A, Figure S5A, B). Indeed, depletion of UPF1 or overexpression of NANOS1 diminished the level of p-ELK1 in A375 cells. Because ERK is an upstream regulator of ELK1 phosphorylation within the MAPK signaling pathway 45, we next assessed ERK phosphorylation under UPF1-depleted or NANOS1-overexpressing conditions. In A375 cells, both UPF1 knockdown and NANOS1 overexpression decreased ERK phosphorylation, which may reduce melanoma tumorigenesis (Figure 7B). It is worth noting that the level of p-PDK1 was not significantly changed. Furthermore, more p-ELK1 was observed in three melanoma cell lines than melanocyte (Figure 7C). In addition, the depletion of NANOS1 reversed the attenuated p-ELK1 levels that resulted from the depletion of UPF1, indicating that UPF1 regulates p-ELK1 levels via NANOS1 (Figure 7D).

Because depletion and overexpression of UPF1 and NANOS1, respectively, reduced p-ELK1, it is conceivable that p-ELK1 reverses the effects of UPF1 and NANOS1 on melanoma tumorigenesis. First, we depleted UPF1 and introduced ELK1 WT or Mut, which was mutated at the phosphorylation site at Serine-383, in three melanoma cell lines. The introduction of ELK1 WT but not ELK1 Mut reversed the effects of depletion of UPF1 on cell growth. Consistently, ELK1 WT erased the effects of NANOS1 overexpression on the growth of melanoma (Figure 7E, Figure S5C, D). Then, we determined the effects of p-ELK1 on migration and invasion ability in the presence or absence of UPF1 and NANOS1 (Figure S6A-D). In support, downregulation and upregulation of UPF1 and NANOS1 efficiently abrogated migration and invasion, which were restored by overexpression of ELK1 WT but not by ELK1 Mut. To further investigate the mechanistic link between NANOS1 and reduced ELK1 phosphorylation, we performed RNA immunoprecipitation (RIP)-seq in A375 cells overexpressing NANOS1-FLAG, using anti-FLAG antibody. We first compared FLAG and NANOS1-FLAG input samples to determine whether NANOS1-FLAG overexpression altered baseline transcript abundance, identifying transcripts that were significantly altered in NANOS1-FLAG input samples compared with FLAG input samples (Figure 7F). We then compared RIP enrichment between NANOS1-FLAG and FLAG control samples and identified a subset of transcripts preferentially enriched in NANOS1-FLAG RIP samples, whose enrichment analysis revealed functional categories related to cell death and signal transduction (Figure 7G, Figure S5E).

To prioritize candidates potentially involved in the NANOS1-mediated regulation of ELK1 phosphorylation, we intersected genes downregulated in NANOS1-FLAG input samples with genes enriched in NANOS1-FLAG RIP samples. This analysis identified 25 overlapping transcripts, including three MAPK signaling-related genes, *MAP2K6, NLK,* and *FOSL2* (Figure 7H). We further selected seven additional MAPK signaling-related transcripts from the NANOS1-FLAG RIP-enriched gene set and validated these candidates by RT-qPCR, indicating that selected transcripts showed markedly higher enrichment in NANOS1-FLAG IP samples compared with FLAG control IP samples (Figure 7I). In addition, heatmap analysis of input FPKM values showed that several candidates (*MAPK1, PPP3R1, CDC42, NRAS, RAP1A, CASP3, LAMTOR3*) were not increased in NANOS1-FLAG input samples, suggesting that their enrichment in NANOS1-FLAG RIP was not simply attributable to higher baseline expression (Figure 7J). Taken together, these results suggest that UMD/NANOS1 axis may associate with MAPK signaling-related transcripts and provide a potential mechanistic link between NANOS1 expression and reduced ERK/ELK1 phosphorylation in melanoma cells.

**UPF1 and NANOS1 regulate the growth of drug-resistant melanoma cells.** One of the challenges in treating melanoma is that it can assume a drug-resistant form 16, 58. Thus, we generated drug-resistant cells to examine the effects of UPF1 and NANOS1 in drug-resistant melanoma cell tumorigenesis. To generate the drug-resistant cell lines, we treated A375 and SK-MEL-28 cell lines, which contained BRAF mutations, with vemurafenib (BRAF inhibitor), and we treated SK-MEL-2, which contained NRAS mutations, with trametinib (MEK inhibitor) until each cell line showed resistance to the inhibitor. Each drug-resistant cell line exhibited strong resistance to increasing amounts of the appropriate inhibitor compared with the parental cell lines, showing elevated levels of the phosphorylated ERK (Figure S7A). However, there were no changes in endogenous UPF1, NANOS1 and miR-101-3p expression (Figure S7B, C). Then, we determined whether UPF1 and NANOS1 retarded cell growth in the drug-resistant cell lines. Depletion of UPF1 and overexpression of NANOS1 also reduced the growth of all three drug-resistant melanoma cell lines, similar to effects in parental cells (Figure 8A). Consistently, downregulated UPF1 and overexpressed NANOS1 in the presence of the vemurafenib or trametinib inhibitor resulted in even greater repression in cell growth than in cells treated with inhibitor only in all three melanoma cell lines (Figure 8B). Consistent with the cell growth results in the case of depletion of UPF1 or overexpression of NANOS1, either UPF1 depletion or NANOS1 overexpression in drug-resistant melanoma cells effectively reduced migration ability in all three cell lines (Figure 8C, D). Consistent with the results observed in parental cells, miR-101-3p mimic increased cell growth and migration, whereas miR-101-3p inhibition suppressed cell growth and migration in drug-resistant cells (Figure S8A, B). As in parental A375 cells, UPF1 depletion or NANOS1 overexpression reduced p-ELK1 levels in A375 drug-resistant cells (Figure S8C). However, no corresponding reduction in ERK phosphorylation was detected in the drug-resistant setting, suggesting that ELK1 phosphorylation may be regulated through ERK-independent or resistance-associated signaling mechanisms in this context. In addition, modulation of p-ELK1 affected cell growth and migration in drug-resistant cells, further supporting the functional relevance of ELK1 phosphorylation in drug-resistant melanoma cells (Figure S8D-F). These findings indicate that the UPF1/NANOS1-related pathway remains functionally relevant in drug-resistant melanoma cells. Collectively, the findings indicate that the UMD/NANOS1 pathway additionally influences cell proliferation in drug-resistant cell lines and is more potent when combined with MAPK inhibitors such as vemurafenib and trametinib.

## Discussion

Melanoma is one of the most aggressive forms of skin cancer; it is driven by various mutations, notably BRAF, NRAS, or NF1 deficiency 10, 11. Despite recent advancements with BRAF and MEK inhibitors in melanoma therapy, drug resistance remains a formidable obstacle, underscoring the urgency for novel treatment modalities. Lower *UPF1* expression was associated with improved overall survival in the SKCM cohort; however, this bulk tumor-based association should be interpreted cautiously and regarded as hypothesis-generating rather than evidence of a melanoma cell-intrinsic tumor-promoting role for UPF1. In line with this clinical observation, our findings demonstrate that UPF1 promotes melanoma cell proliferation, migration, and invasion in both *in vitro* and *in vivo* models. Specifically, UPF1 depletion suppressed the growth of melanoma cells harboring BRAF or NRAS mutations and impaired their migratory and invasive capacities by inducing G1-phase cell-cycle arrest (Figures 1 and 2). Although UPF1 has been reported to function as a tumor suppressor in several cancer types 32-34, 36, its tumor-promoting role in melanoma may reflect the context-dependent nature of UPF1-mediated RNA surveillance. Because the biological effects of UPF1 are determined by the identity and function of its target transcripts, differences in transcriptomic and miRNA landscapes among tumor types may lead to distinct or even opposing phenotypic outcomes. Indeed, transcriptomic profiling of UPF1-depleted melanoma cells unveiled a notable increase in the expression of another posttranscriptional regulatory gene, NANOS1, consistently observed across all melanoma cell lines. Subsequent mRNA stability analyses revealed direct UPF1-mediated regulation of NANOS1 expression. Notably, these findings were corroborated through patient-derived single-cell RNA-seq analysis, suggesting potential clinical relevance (Figure 3). Consistently, public TCGA database showed higher *UPF1* expression in melanoma patients than in normal samples, whereas *NANOS1* expression showed an opposite tendency. Therefore, these public transcriptomic analyses provide supportive context for the UPF1/NANOS1 relationship, but should not be interpreted as definitive evidence of causality. We next investigated the role of NANOS1 and found that it suppresses melanoma cell proliferation, migration, and invasion by inducing cell-cycle arrest (Figure 4). Combined depletion of UPF1 and NANOS1 restored melanoma cell proliferation, further supporting the functional interplay between these two factors. These findings were further validated *in vivo* (Figure 5). In contrast to previous reports describing pro-migratory and pro-invasive functions of NANOS1 in other cancer types, our findings support a suppressive role for NANOS1 in melanoma, highlighting its tumor-type- and context-dependent functions.

While UPF1 is known for its role in nonsense-mediated mRNA decay (NMD) within the exon junction complex 59, 60, our study explored its potential function in regulation of NANOS1, a single-exon gene. We hypothesized SMG7-dependent regulation of NANOS1 by UPF1, known as UMD, via its 3'UTR. Through reporter assays, we delineated a UPF1-regulated region within the *NANOS1* 3'UTR 850-nt. Subsequent SMG7 depletion reflected the UPF1 effect, upregulating *NANOS1* mRNA levels. Moreover, miR-101-3p was identified as a regulator of *NANOS1* expression, indicating UPF1/SMG7-dependent miRNA-mediated regulation (UMD) (Figure 6). Consistently, miRNA-101-3p mimic and inhibitor increased and decreased the melanoma cell growth and migration, respectively. Further elucidation of the cellular signaling cascades revealed the involvement of p-ELK1 (Figure 7). Consistent with this model, UPF1 depletion and NANOS1 overexpression reduced ERK and ELK1 phosphorylation in parental A375 cells, suggesting that the UPF1/NANOS1 axis may suppress melanoma phenotypes, at least in part, through attenuation of MAPK/ERK/ELK1 signaling. NANOS1-FLAG RIP-seq and subsequent RT-qPCR validation further identified a subset of NANOS1-associated transcripts involved in MAPK signaling and signal transduction. Together, these findings suggest that NANOS1 may influence MAPK-related RNA networks, providing a plausible mechanistic link between NANOS1 expression and reduced ERK/ELK1 signaling in melanoma. However, miR-101-3p is likely to regulate multiple transcripts; therefore, some of its effects on melanoma growth and MAPK/ELK1-related signaling may be mediated through additional targets independently of NANOS1. Accordingly, we interpret miR-101-3p as an upstream regulatory component contributing to NANOS1 mRNA regulation, rather than as the sole mediator of the downstream phenotypic and signaling effects associated with the UPF1/NANOS1 axis. Given the clinical challenge of drug resistance in melanoma therapy 16, 58, we evaluated the impact of UPF1 and NANOS1 dysregulation on drug-resistant melanoma cells. Notably, downregulation of UPF1 and upregulation of NANOS1 enhanced the efficacy of vemurafenib and trametinib. In addition, ERK phosphorylation was not decreased in A375 drug-resistant cells, suggesting that constitutive MAPK/ERK activation or resistance-associated signaling rewiring may mask the ERK-regulatory effect of the UPF1/NANOS1 axis in the drug-resistant context (Figure 8).

In summary, our study unveils NANOS1 as a direct target of UPF1, shedding light on the intricate regulatory mechanisms governing melanoma pathogenesis. By elucidating the interplay between UPF1, NANOS1, and miRNA-mediated pathways, we offer novel insights into the molecular underpinnings of melanoma tumorigenesis (Figure 8E). These findings not only expand our understanding of melanoma biology, but also underscore the intricacy of gene regulatory pathways in cancer progression. Our comprehensive experimental approach encompassing both *in vitro* and *in vivo* analyses underscores the clinical relevance of these regulatory mechanisms in the context of melanoma therapy. By delineating the roles of the UMD pathway through UPF1 and NANOS1 in melanoma progression and in drug-resistant melanoma cell lines, our study lays the groundwork for targeted therapeutic strategies to disrupt these regulatory pathways. Ultimately, these findings hold promise for the advancement of precision medicine approaches in the treatment of melanoma, offering new avenues for therapeutic intervention and improved patient outcomes.

## Supplementary Material

Supplementary figures and tables.

## Figures and Tables

**Figure 1:**
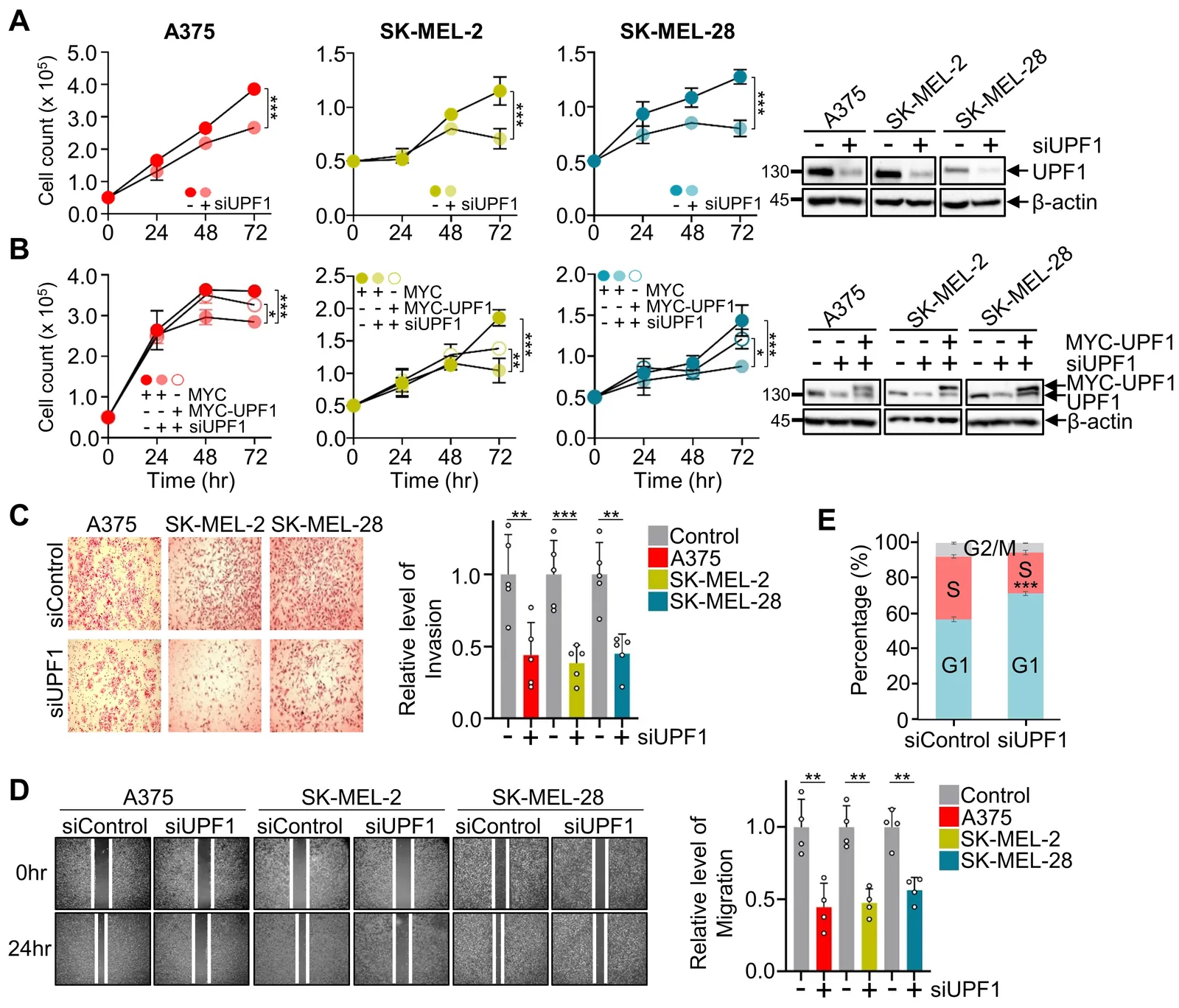
** UPF1 regulates the growth of melanoma cells. (A)** UPF1 was depleted by siRNA in the indicated three melanoma cell lines, and cell growth was measured by cell counting over 72 hr. Each UPF1-depleted cell count was normalized by control cells and plotted for each condition (n=3). WB was performed to evaluate the UPF1 level. **(B)** Similar to A, however, downregulated UPF1 was rescued by siRNA-resistant UPF1 expression (n=3). **(C, D)** Cell invasion assay (n=5) (C) and migration assay (n=4) (D) were performed using the indicated UPF1-depleted melanoma cell lines. Relative levels of invasion **(**C) and migration (D) were quantified. **(E)** The cell cycle distribution in UPF1-depleted A375 cells was evaluated by flow cytometry (n=3). Statistical analysis: two-way ANOVA (A,B), Student's t-tests (C,D) and Bonferroni correction (E). * P ≤ 0.05; ** P ≤ 0.01; *** P ≤ 0.001. Each error bar represents the mean ± standard deviation.

**Figure 2:**
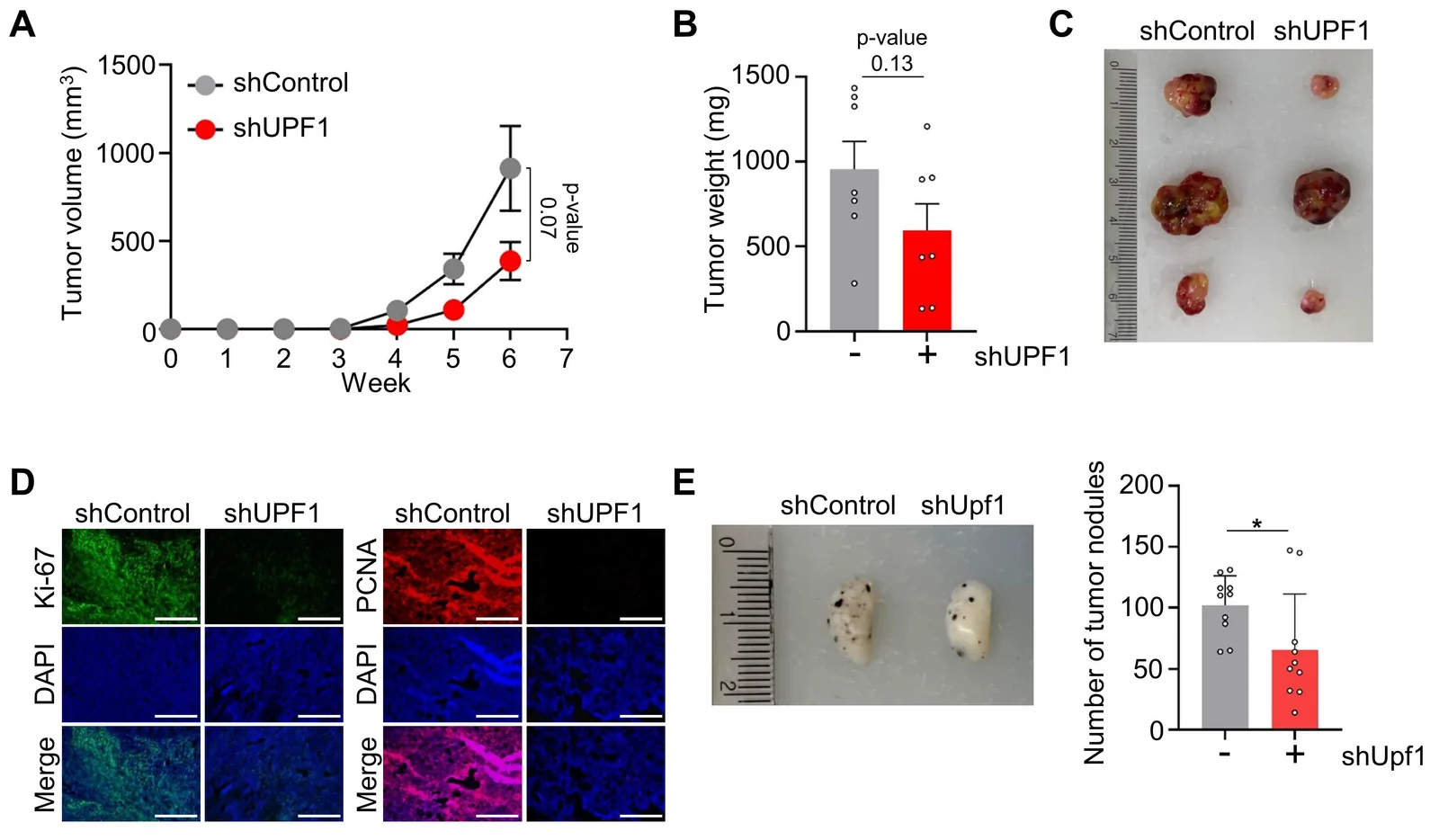
** Depletion of UPF1 attenuated tumor growth *in vivo*. (A)** The volume of tumors formed in nude mice grafted subcutaneously with UPF1-depleted A375 cells was monitored weekly for 6 weeks (n=8). **(B)** Tumors were weighed after removal. **(C)** Representative tumor images from grafted mice 6 weeks after cell implantation. **(D)** Immunofluorescence (Scale bar = 250µm) was performed for Ki-67 and PCNA in xenografted mice tumors. **(E)** Representative lung image from Upf1-depleted B16F10 melanoma-injected mice (n=10). The number of colonies in each lung was counted. Statistical analysis: two-way ANOVA (A) and Student's t-tests (B, E). Exact p-values are indicated for (A) and (B), and * p ≤ 0.05 denotes statistical significance in (E). Each error bar represents the mean ± standard deviation.

**Figure 3:**
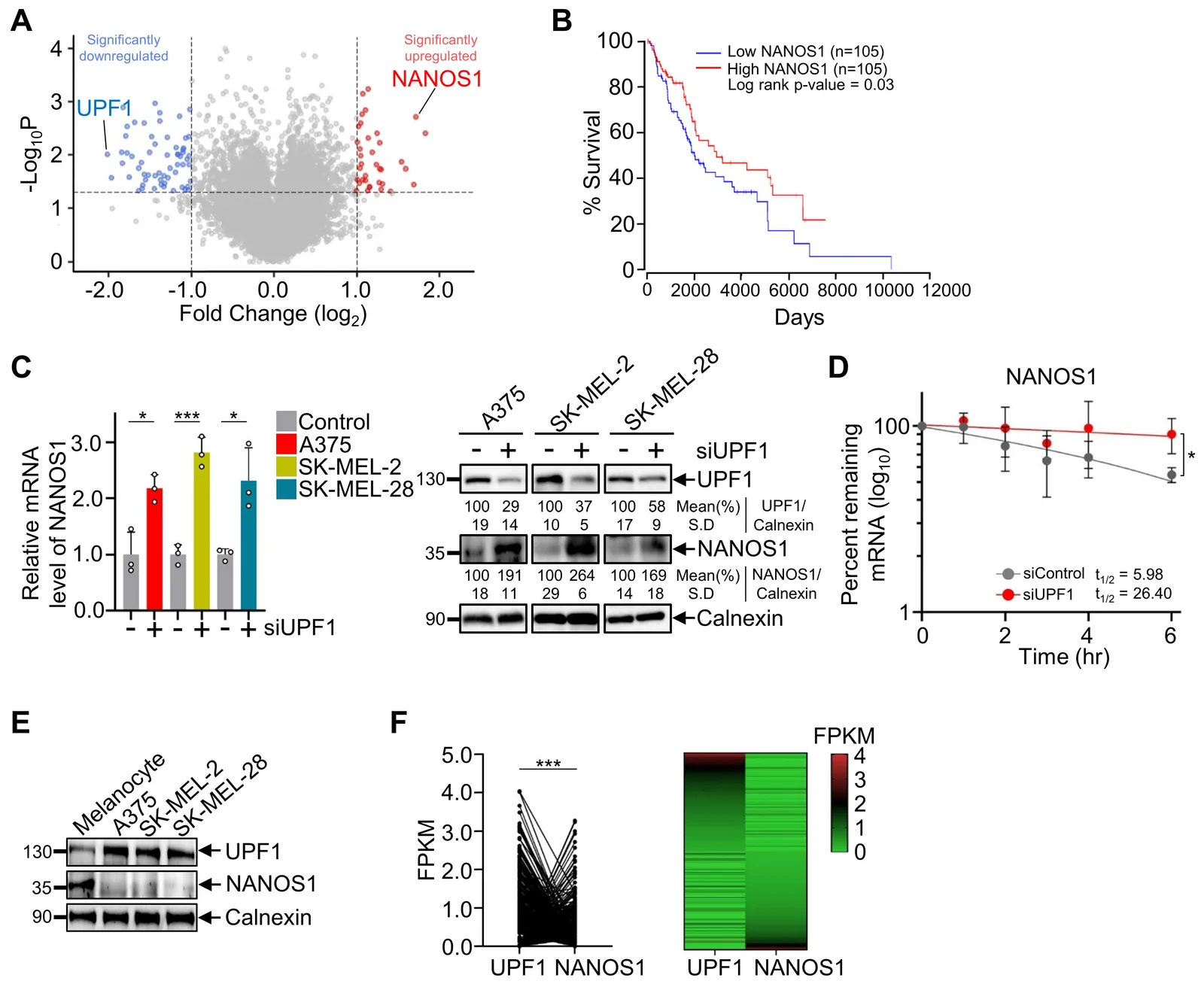
** UPF1 posttranscriptionally regulated NANOS1 level. (A)** Volcano plot showing the log2-fold change in expression between UPF1-depleted and control siRNA-transfected A375 cells. The horizontal line indicates the significance threshold (n=2). Two different siUPF1 combinations were employed. **(B)** Kaplan-Meier curves display the overall survival of patients with low *NANOS1* expression vs. high *NANOS1* expression. Data sets were obtained from Oncolnc. Database.** (C)** RT-qPCR and WB were performed to evaluate the levels of NANOS1 upon UPF1-depletion in the indicated cell lines (n=3). **(D)** A375 cells transfected with siUPF1 were treated with DRB. The level of *NANOS1* mRNA was measured at the indicated time point. The relative level of mRNA was normalized to that of *GAPDH* mRNA. **(E)** The levels of endogenous UPF1 and NANOS1 expression in the indicated cells were measured by WB**. (F)** The public melanoma patient single-cell RNA-seq dataset was analyzed for comparison of *UPF1* with *NANOS1* expression 61. The FPKM values of the top 20% of cells with high expression of *UPF1* or *NANOS1* among 1257 malignant cells were indicated as matching (left panel) and heatmap (right panel). Statistical significance was calculated from at least three independent experiments using Student's t-test. * p ≤ 0.05; *** p ≤ 0.001. Each error bar represents the mean ± standard deviation.

**Figure 4:**
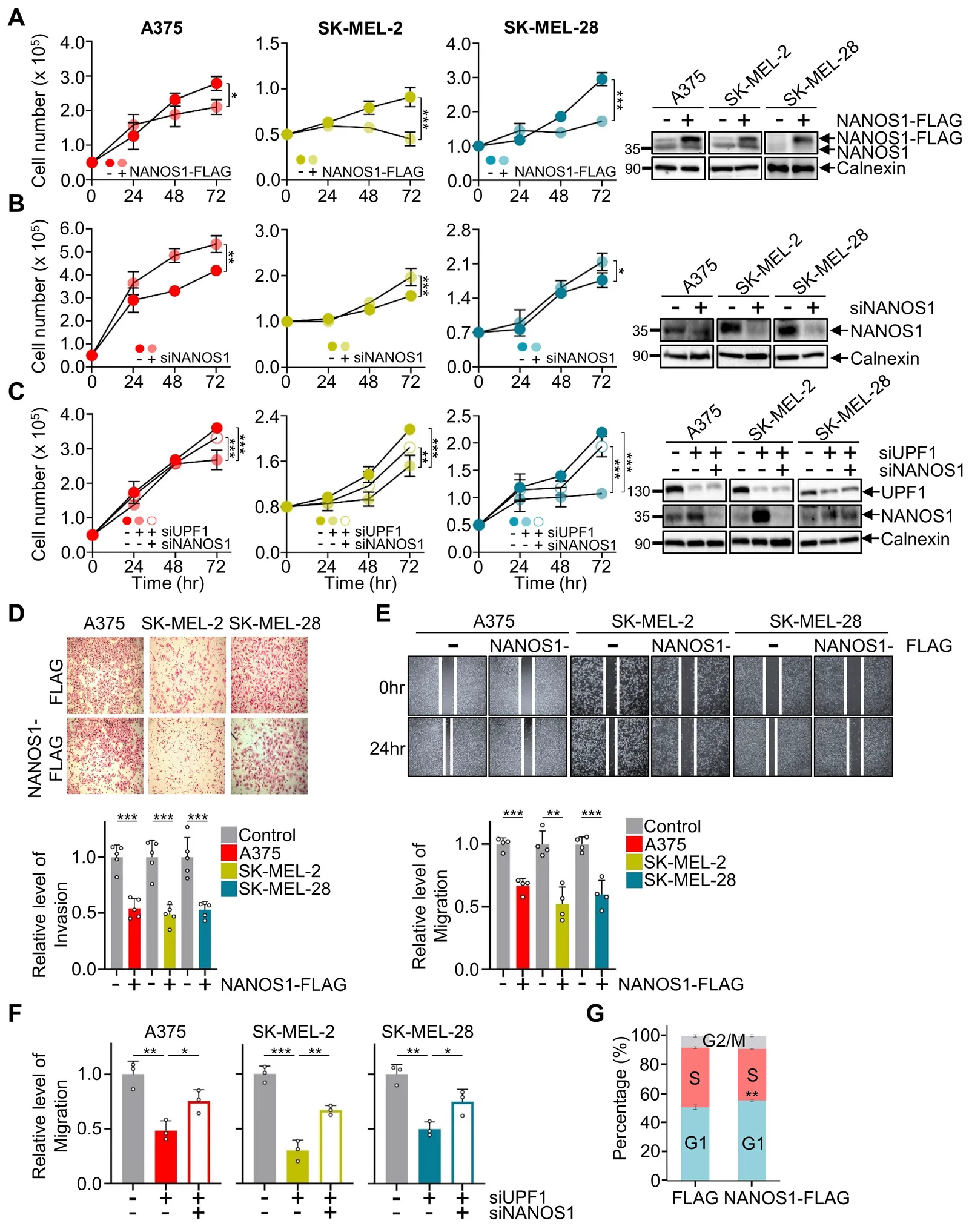
** Overexpression of NANOS1 reduces melanoma tumorigenesis. (A-C)** C-terminally FLAG-tagged NANOS1 (NANOS1-FLAG) (A), siNANOS1 (B), or both siUPF1 and siNANOS1 (C) were transiently transfected into the indicated melanoma cell lines, and cell growth was measured by cell counting over 72 hr. Each cell count was normalized by control cells and plotted for each condition (n=3). WB was employed to evaluate the indicated protein levels. **(D, E)** Cell invasion assay (D) and migration assay (E) were performed using the NANOS1-FLAG expressing melanoma cell lines. Relative levels of invasion (n=5) (D) and migration (n=4) (E) were quantified. **(F)** Cell migration assays were performed using melanoma cell lines depleted of UPF1 and/or NANOS1, and the results were quantified (n=3). **(G)** The cell cycle distribution in NANOS1-FLAG expressing A375 cells was evaluated by flow cytometry (n=3). Statistical analysis: two-way ANOVA (A-C), Student's t-tests (D-F) and Bonferroni correction (G). * p ≤ 0.05; ** p ≤ 0.01; *** p ≤ 0.001. Each error bar represents the mean ± standard deviation.

**Figure 5:**
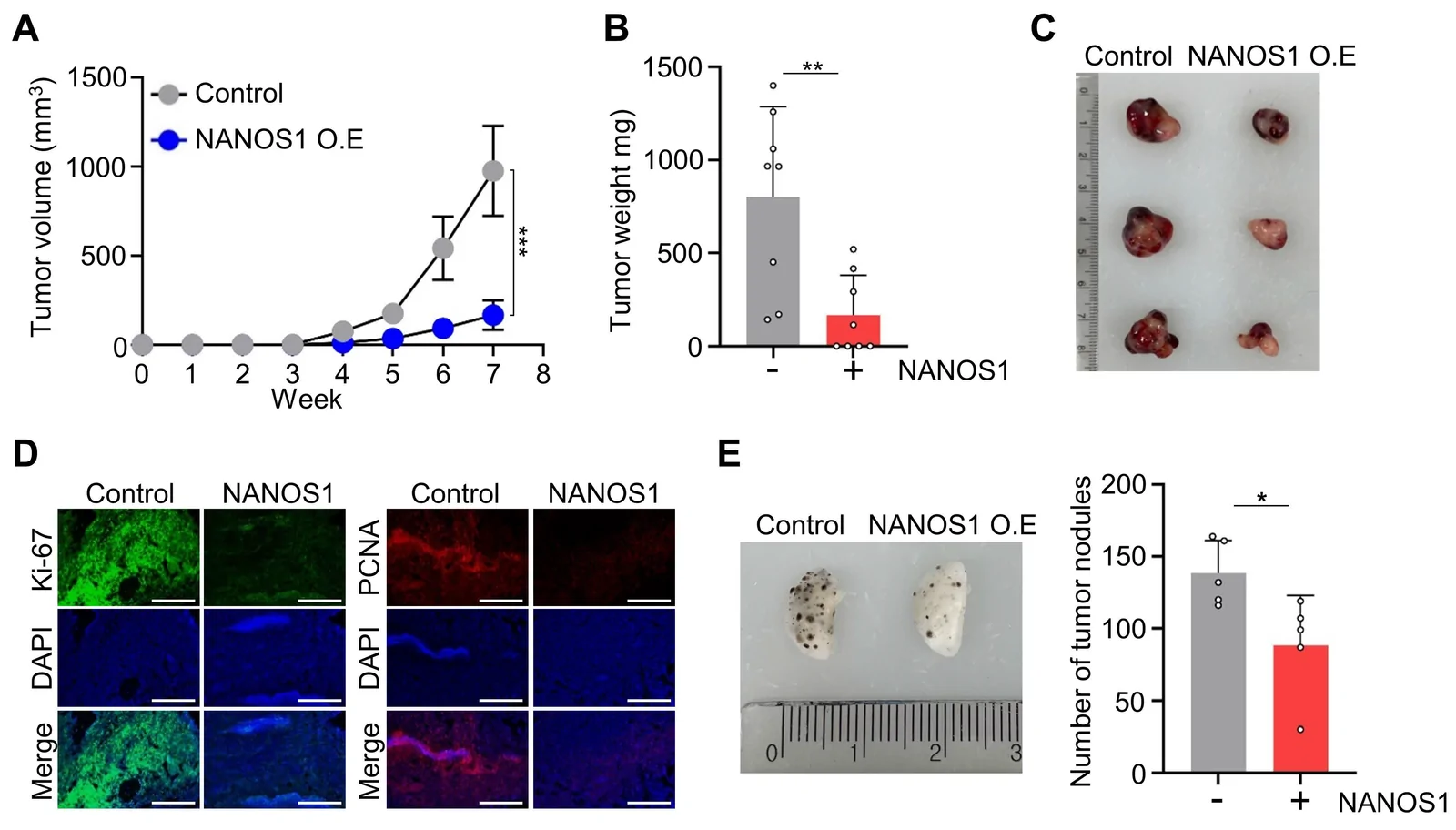
** NANOS1 reduces tumor growth *in vivo*. (A)** The volume of tumors formed in nude mice grafted subcutaneously with NANOS1-FLAG-overexpressing A375 cells was monitored weekly for 7 weeks (n=8). **(B)** Tumors were weighed after removal. **(C)** Tumors from grafted mice 7 weeks after cell implantation. **(D)** Immunofluorescence (Scale bar = 250µm) was performed for Ki-67 and PCNA in xenografted tumors in nude mice. **(E)** Representative lung image from NANOS1-FLAG-overexpressing B16F10 melanoma-injected mice. The number of colonies in each lung was counted (n=5). Statistical analysis: two-way ANOVA (A) and Student's t-tests (E). * p ≤ 0.05; ** p ≤ 0.01; *** p ≤ 0.001. Each error bar represents the mean ± standard deviation.

**Figure 6:**
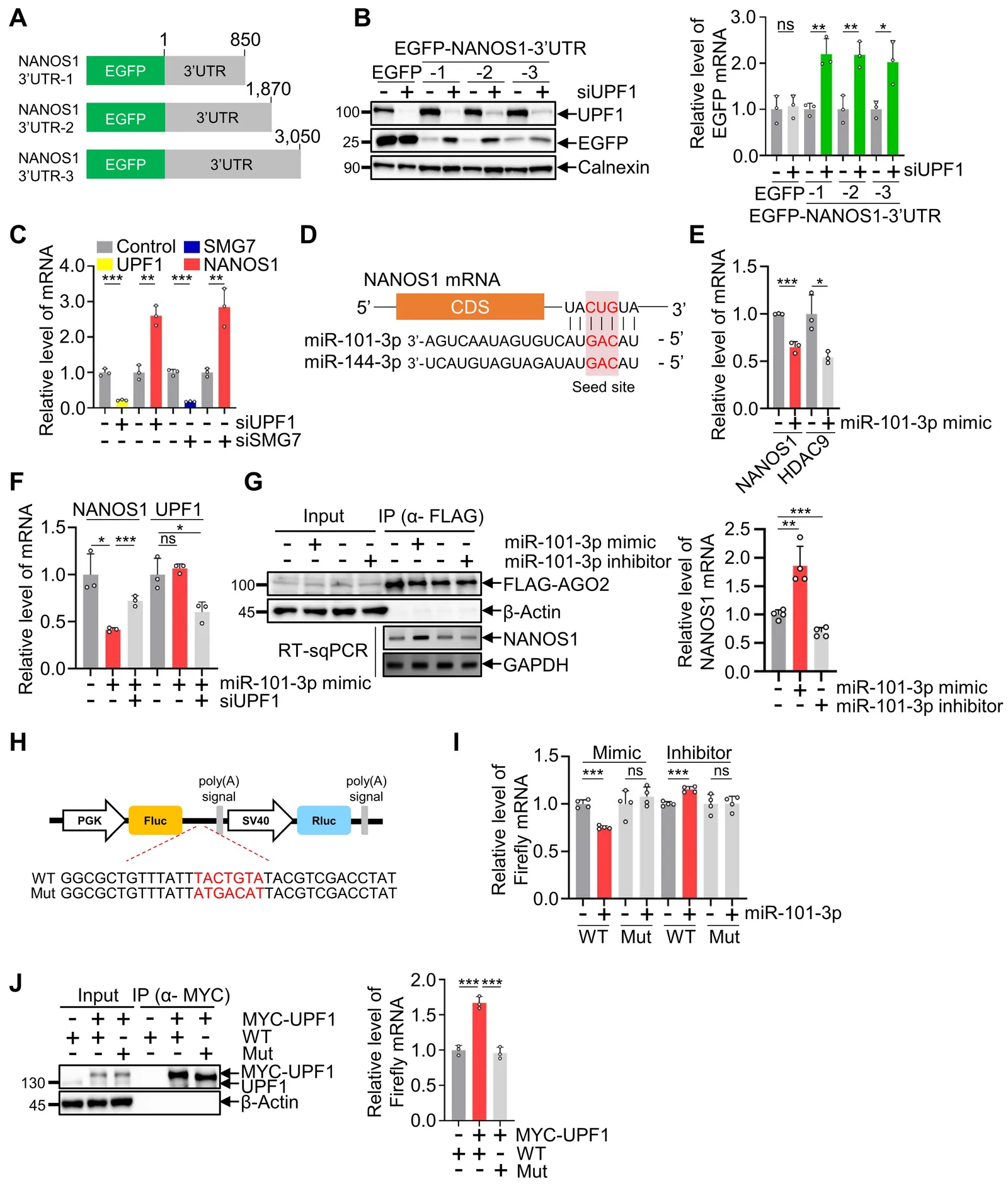
** UPF1 targets the 3'UTR of *NANOS1* via the UMD pathway. (A)** The representative constructs containing EGFP with various length of *NANOS1* 3'UTR. **(B)** A375 cells that were transfected with the indicated EGFP-NANOS1 3'UTR in the presence or absence of UPF1 were employed for WB and RT-qPCR (n=3). **(C)** The indicated transcripts were measured by RT-qPCR using UPF1- or SMG7-depleted A375 cells (n=3). **(D)** Schematic representation of miR-101-3p and miR-144-3p seed site alignment in 3'UTR of *NANOS1*. **(E)** The levels of indicated transcripts were measured by RT-qPCR using miR-101-3p mimic-transfected A375 cells (n=3). **(F)** A375 cells that were transfected with miR-101-3p mimic and/or siUPF1 were employed for RT-qPCR to evaluate the levels of *NANOS1* (n=3). **(G)** FLAG-AGO2 was transfected into A375 cells in the presence of miR-101-3p mimic or inhibitor, and IP was performed using anti-FLAG beads. WB, RT-qPCR, and semi RT‒qPCR (RT-sqPCR) were employed to evaluate IP eluates and the target RNA (n=4). **(H)** Schematic representation of a dual luciferase construct containing putative wild-type (WT) or mutant (Mut) seed sites in *NANOS1* 3'UTR. The putative WT binding sequences and CTG-to-GAC mutant in Mut were indicated. **(I)** WT or Mut miR-101-3p binding sites expressing reporter plasmids were co-transfected with miR-101-3p mimic or inhibitor in A375 cells. The relative amount of FLuc mRNA normalized to the level of RLuc mRNA was measured by RT-qPCR (n=4). **(J)** Similar to (I), however, coimmunoprecipitated WT or Mut reporter RNA with MYC-UPF1 were quantified by RT-qPCR using anti-MYC immunoprecipitation (n=3). Statistical significance was calculated from at least three independent experiments using Student's t-test. * p ≤ 0.05; ** p ≤ 0.01; *** p ≤ 0.001; ns, not significant. Each error bar represents the mean ± standard deviation.

**Figure 7:**
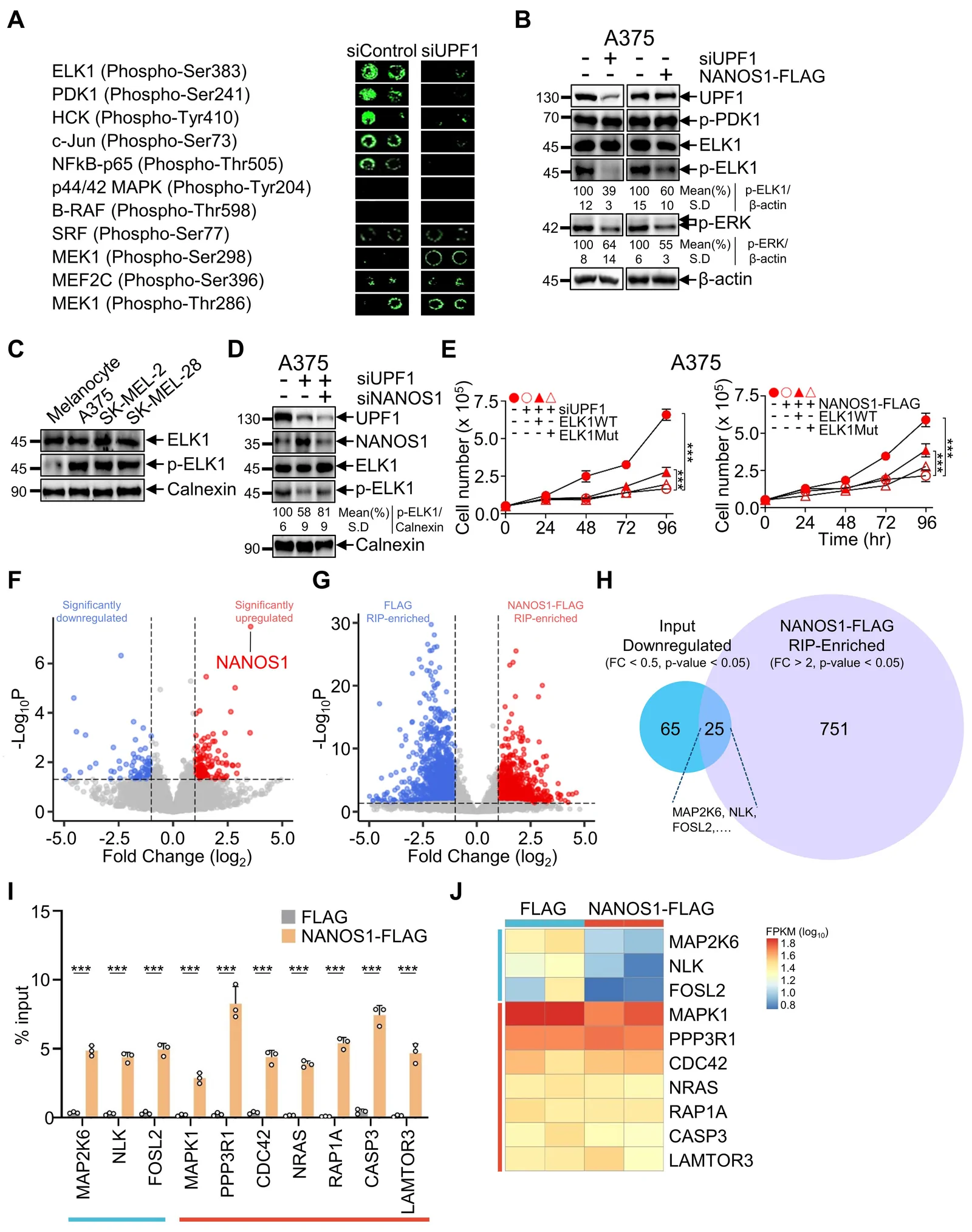
** The UPF1/miR-101-3p/NANOS1 axis regulated melanoma tumorigenesis through phosphorylated ERK/ELK1. (A)** Representative phospho-antibody array images showing phosphorylation changes in UPF1-depleted A375 cells. **(B)** Quantification of phospho-ELK1 and phospho-ERK levels. Band intensities were normalized to the indicated loading control and are shown relative to control cells (n=3). **(C)** The levels of endogenous p-ELK1 expression in the indicated cells (melanocyte or melanoma cells) were observed by WB. **(D)** The levels of endogenous p-ELK1 expression with UPF1 and/or NANOS1 depleted A375 cells were observed by WB (n=3). **(E)** A375 cells were transfected with ELK1 WT or ELK1 Mut in UPF1-depleted cells or NANOS1-FLAG overexpressing cells. Cell growth was monitored for 96 hr by cell counting. Each cell count was normalized by control cells and plotted for each condition (n=3). (F) Volcano plot showing the log2-fold change in expression between NANOS1-FLAG input and control FLAG input from A375 cells. The horizontal line indicates the significance threshold (n=2). (G) Volcano plot showing differential RIP enrichment between NANOS1-FLAG and control FLAG-RIP samples. The horizontal dashed line indicates the significance threshold (n=4). (H) Venn diagram showing the overlap between transcripts significantly downregulated in NANOS1-FLAG input samples and those significantly enriched in NANOS1-FLAG RIP samples. Downregulated and RIP-enriched transcripts were defined using fold-change thresholds of < 0.5 and >2.0, respectively. Numbers in each section indicate the number of transcripts. FC, fold change. (I) RT-qPCR validation of the indicated MAPK signaling-related transcripts identified from the NANOS1-FLAG RIP eluates. The left three genes (*MAP2K6, NLK, FOSL2*) represent transcripts downregulated in NANOS1-FLAG input samples and enriched genes in NANOS1-FLAG RIP samples. The right seven genes (*MAPK1, PPP3R1, CDC42, NRAS, RAP1A, CASP3, LAMTOR3*) represent transcripts enriched only in NANOS1-FLAG RIP samples. (J) Heatmap showing input log10(FPKM) values of selected MAPK signaling-related candidate transcripts. Genes are arranged in the same order as in the RT-qPCR panel, with the upper three and lower seven representing the two candidate groups. Statistical analysis: two-way ANOVA (E) and Student's t-tests (I). *** P ≤ 0.001. Each error bar represents the mean ± standard deviation.

**Figure 8:**
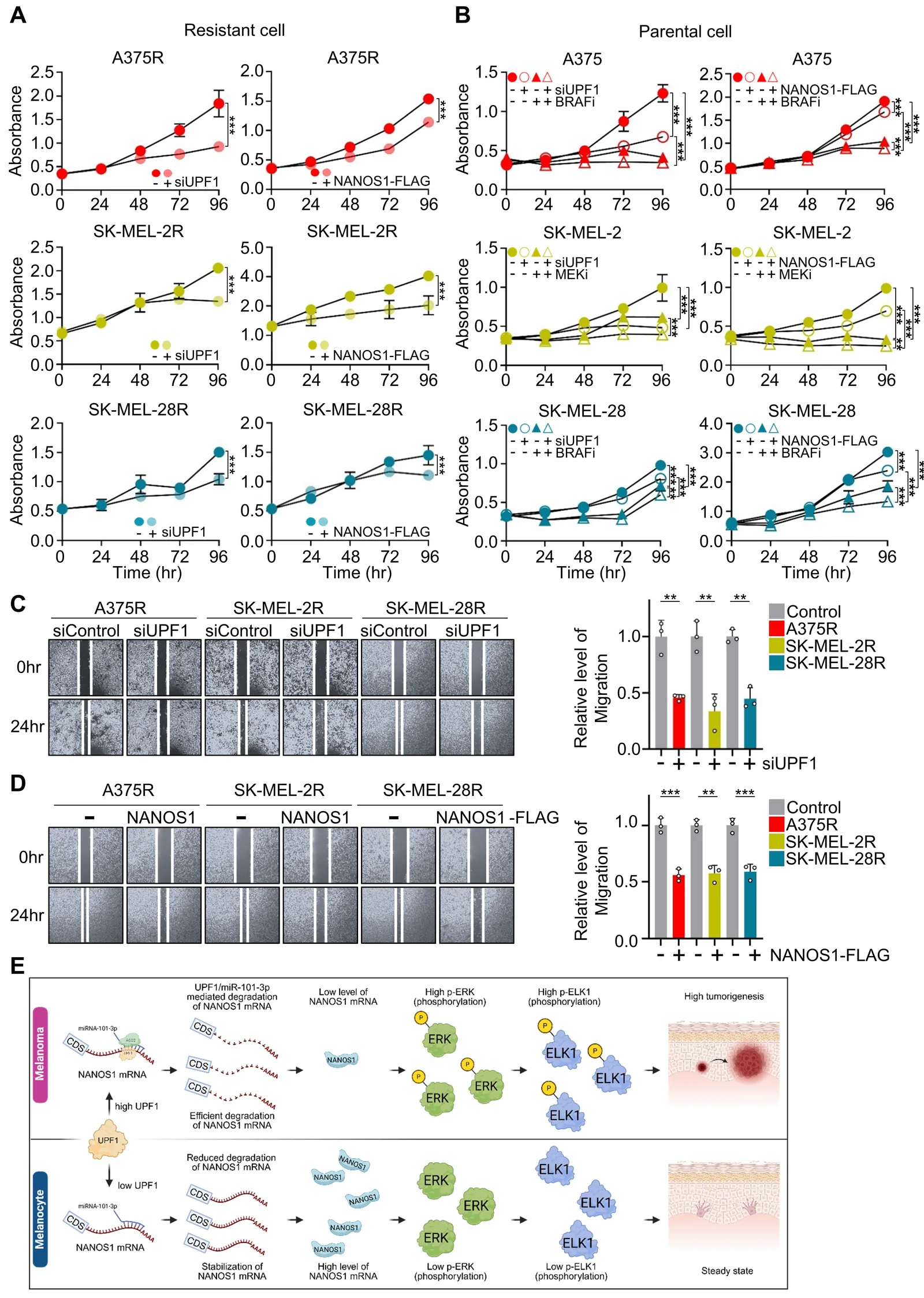
** UPF1 and NANOS1 regulate the proliferation of both drug-treated and drug-resistant melanoma cells. (A)** The indicated BRAF inhibitor- (A375R and SK-MEL-28R) or MEK inhibitor- (SK-MEL-2R) resistant melanoma cell lines were transfected with siUPF1 or NANOS1-FLAG. The cell growth was monitored for 96 hr by CCK8. Each well was normalized by control cells and plotted for each condition (n=3). **(B)** Same as (A), however, parental cells were used in the presence or absence of BRAF or MEK inhibitor (n=3). **(C, D)** Cell migration assays were performed using drug-resistant melanoma cell lines in the depletion of UPF1 (C) or overexpression of NANOS1-FLAG (D), and results were quantified (n=3). **(E)** Suggested model. Statistical analysis: two-way ANOVA (A, B) and Student's t-tests (C, D). * p ≤ 0.05; ** p ≤ 0.01; *** p ≤ 0.001. Each error bar represents the mean ± standard deviation.
