# Supplementary material for: UPF1-mediated mRNA decay promotes melanoma tumorigenesis through miR-101-3p-dependent destabilization of NANOS1
Source: Int J Biol Sci. 2026 Sep 24;22(15):8492–510. doi: 10.7150/ijbs.134804 (PMC13685752; doi:10.7150/ijbs.134804)
Supplement: Supplementary file 1 [file ijbsv22p8492s1.pdf]

Supplementary figure legends

Figure S1 (A) Kaplan-Meier curves display overall survival of the various cancer types with low *UPF1* expression vs. high *UPF1* expression in the Oncolnc database (www.oncolnc.org). (B) Box plots display the expression levels of *UPF1* in tumor samples categorized by clinical stage, based on The Cancer Genome Atlas (TCGA) dataset. (C) Representative IHC images showing UPF1 expression (scale bar = 100μm). Statistical significance was calculated using Student's t-test. * $p \leq 0.05$; ** $p \leq 0.01$. Each error bar represents the mean ± standard deviation.

Figure S2 (A) UPF1 was depleted by siRNA in the metastatic A375 (A375SM) cells, and growth was measured by cell counting over 72 hr. UPF1-depleted cell count was normalized by control cells and plotted for each condition (n=3). WB was performed to evaluate the UPF1 level. (B) Schematic representation of a siRNA-resistant UPF1 construct. (C) (Corresponding to Figure 1E) Flow cytometry was performed to detect the levels of 7-AAD and BrdU in UPF1-depleted A375 cells. (D) Flow cytometry was performed to detect the levels of Annexin-V and PI in UPF1-depleted A375 cells (n=3). WB was performed. (E) (Corresponding to Figure 2A-C) Representative images of tumors formed in nude mice grafted subcutaneously with UPF1-depleted A375 cells. (F) WB and RT-qPCR were performed to evaluate the levels of UPF1 from xenografted tumors. mRNA levels were normalized to that of *GAPDH* mRNA. (G) Mouse Upf1 was depleted by siRNA in the B16F10 mouse melanoma cell line, and cell growth was measured by cell counting over 72 hr. WB was performed to evaluate the Upf1 level (n=3). (H) MYC-UPF1 was transiently transfected into the MEL-ST cells, and cell growth was measured by cell counting over 72 hr. WB was performed to evaluate the UPF1 level (n=3). Statistical analysis: two-way ANOVA (A, G, H) and Student's t-tests (D, F). *** $p \leq 0.001$; ns, not significant. Each error bar represents the mean ± standard deviation.

Figure S3 (A) (Corresponding to Figure 3A) Venn diagram indicated commonly upregulated transcripts (*NANOS1* and *LINC01546*, $p < 0.05$) by 2-fold upon UPF1-depletion from three independent RNA-seqs. UPF1 in A375 cells were depleted using UPF1 CDS (coding sequence)-targeting siRNA (n=2), UPF1 3'UTR-targeting siRNA (n=2), or both targeting siRNAs (CDS and 3'UTR) (n=2). (B) Box plots display the expression levels of *NANOS1* in tumor samples categorized by clinical stage, based on The Cancer Genome Atlas (TCGA) dataset. (C) (Corresponding to Figure 2A-C) WB and RT-qPCR were performed to evaluate the levels of NANOS1 from xenografted tumors. mRNA levels were normalized to that of *GAPDH* mRNA. (D) (Corresponding to Figure 3D) The level of *GAPDH* and *β-actin* mRNA was measured at the indicated time point. The relative level of mRNA was normalized to that of *18s* rRNA (n=3). (E) Immunofluorescence microscopy (scale bar = 100μm) showed cell proliferating marker Ki-67 in NANOS1-FLAG-overexpressing A375 cells. Nuclei were stained with DAPI (blue). The relative mean fluorescence intensity (MFI) was measured (n=3). (F) Same as (E), but UPF1-depleted and/or NANOS1-depleted A375 cells were employed (n=3). (G) (Corresponding to Figure 4F) Migration assay were performed in the presence or absence of UPF1 and NANOS1. (H) (Corresponding to Figure 4G) Flow cytometry was performed to detect the levels of 7-AAD and BrdU in NANOS1-FLAG-overexpressing A375 cells. (I) Flow cytometry was performed to detect the levels of Annexin-V and PI in NANOS1-FLAG-overexpressing A375 cells (n=3). WB was performed to evaluate the levels of the indicated proteins. Statistical significance was calculated from at least three independent experiments using Student's t-test. * $p \leq 0.05$; ns, not significant. Each error bar represents the mean ± standard deviation.

Figure S4 (A) (Corresponding to Figure 5A-C) Representative images of tumors formed in nude mice grafted subcutaneously with NANOS1-FLAG-overexpressing A375 cells. (B) WB were performed to evaluate the level of NANOS1 from xenografted tumors. (C) (Corresponding to Figure 6A) Read mapping on *NANOS1* by long-read sequencing (Nanopore sequencing) and short-read sequencing (NGS). (D) The relative amounts of miR-101-3p and miR-144-3p in skin were obtained from TissueAtlas (https://ccb-web.cs.uni-saarland.de/tissueatlas2). (E) Conservation of the putative miR-101-3p binding sequences in *NANOS1* 3'UTR through mammals. (F) Relative miR-101-3p levels in UPF1-depleted melanoma cells, as measured by stem-loop RT-qPCR (n=4). (G) The three leftmost lanes analyzed 2-fold dilutions of RT-sqPCR products using in vitro transcribed *NANOS1* RNA or RT-sqPCR products using *NANOS1* mRNA from A375 cells were loaded onto an agarose gel to determine the mole number of *NANOS1* per A375 cell. The sqPCR products were visualized using EtBr staining. The mole number of in vitro transcribed *NANOS1* RNA was used for the standard curve. (H, I) Cell growth (n=4) (H) and migration assay (n=4) (I) were performed using the miR-101-3p mimic or inhibitor. Cell growth was measured by CCK8 over 120 hr. Statistical analysis: Student's t-tests (F, I) and two-way ANOVA (H). *** $p \leq 0.001$; ns, not significant. Each error bar represents the mean ± standard deviation.

Figure S5 (A) (Corresponding to Figure 7A) Heatmap showing the log2 phosphorylation intensity changes of genes associated with MAPK signaling. (B) A total of 34 proteins whose phosphorylation intensity was significantly changed by more than 2-fold was employed for KEGG analysis. (C) (corresponding Figure 7E) The levels of indicated proteins were observed by WB. (D) The indicated melanoma cell lines were transfected with ELK1 WT or ELK1 Mut in the presence or absence of UPF1 and overexpression of NANOS1-FLAG, and cell growth

was monitored for 96 hr by cell counting. Each cell count was normalized by control cells and plotted for each condition (n=3). (E) NANOS1-FLAG RIP-enriched transcripts were employed for enrichment analysis. Statistical significance was calculated from at least three independent experiments using two-way ANOVA. *** $p \leq 0.001$. Each error bar represents the mean ± standard deviation.

Figure S6 (A, B) Cell migration assay were performed using the melanoma cell lines overexpressing ELK1 WT or ELK1 Mut in the presence or absence of UPF1 or overexpressed NANOS1-FLAG. Relative levels of migration were quantified. (C, D) Similar to (A, B), but an invasion assay was performed. Statistical significance was calculated from at least three independent experiments using Student's t-test. * $p \leq 0.05$; ** $p \leq 0.01$; *** $p \leq 0.001$. Each error bar represents the mean ± standard deviation.

Figure S7 (A) Generation of drug-resistant melanoma cell lines. The indicated drug-resistant cell lines (A375R, SK-MEL-2R, and SK-MEL-28R) and parental cells were treated with various concentrations of BRAF inhibitor (vemurafenib) or MEK inhibitor (trametinib) for 48 hr, and cell viability was measured (n=3). IC50 values were determined. WB was performed to evaluate the drug response. (B) The levels of endogenous UPF1 and NANOS1 in the indicated cell (parental or resistant cells) were observed by WB. (C) The levels of endogenous miR-101-3p in the indicated cell (parental or resistant cells) were observed by RT-qPCR (n=4). Statistical significance was calculated from at least four independent experiments using Student's t-test. ns, not significant. Each error bar represents the mean ± standard deviation.

Figure S8 (A, B) Cell growth (n=4) (A) and migration assay (n=4) (B) were performed using the miR-101-3p mimic or inhibitor. Cell growth was measured by CCK8 over 120 hr. (C) Quantification of phospho-ELK1 and phospho-ERK levels in drug-resistant A375 cells. Band intensities were normalized to the indicated loading control and are shown relative to control cells (n=3). (D) ELK1 WT or ELK1 Mut was transiently transfected in UPF1-depleted cells or NANOS1-FLAG overexpressing drug-resistant cells. Cell growth was monitored for 96 hr by cell counting. Each cell count was normalized by control cells and plotted for each condition (n=3). (E-F) Cell migration assay were performed using the drug-resistant melanoma cell lines overexpressing ELK1 WT or ELK1 Mut in the presence or absence of UPF1 (E) or overexpressed NANOS1-FLAG (F) (n=3). Statistical analysis: two-way ANOVA (A, D) and Student's t-tests (E, F). * $p \leq 0.05$; ** $p \leq 0.01$; *** $p \leq 0.001$. Each error bar represents the mean ± standard deviation.

Revised Supplementary Figure 1

A

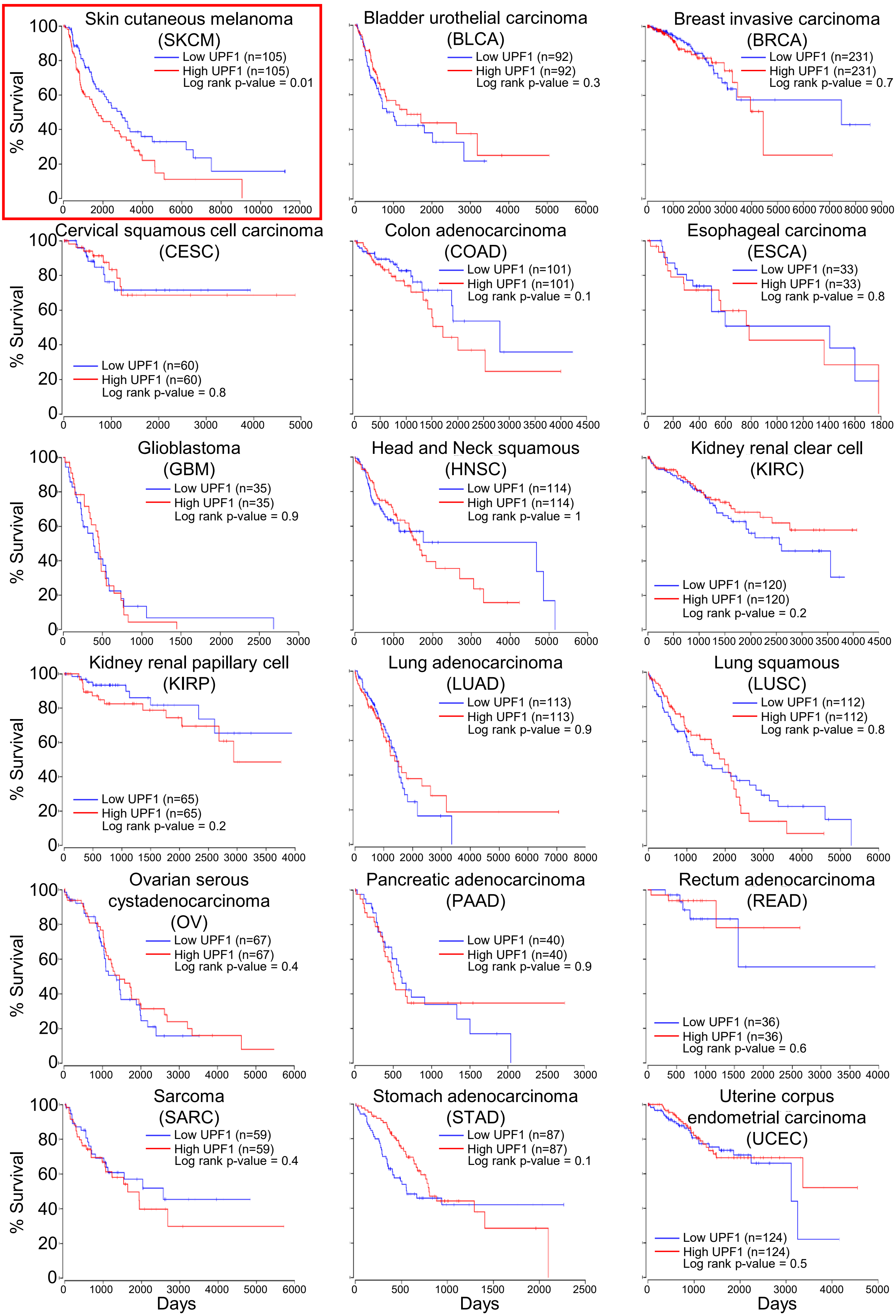

Revised Supplementary Figure 1

B

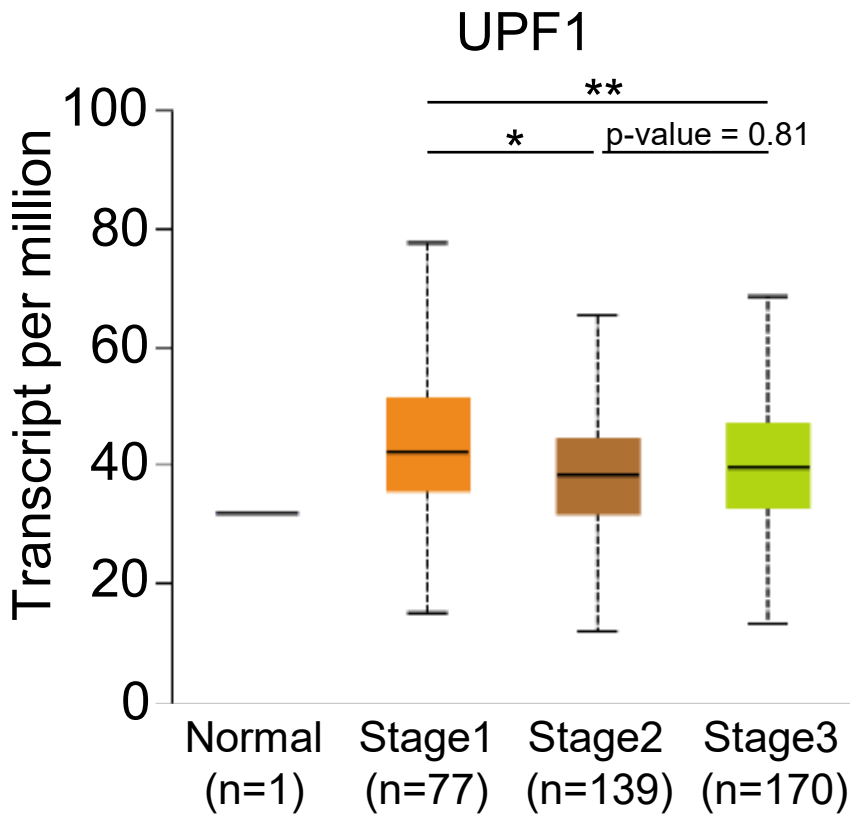

C

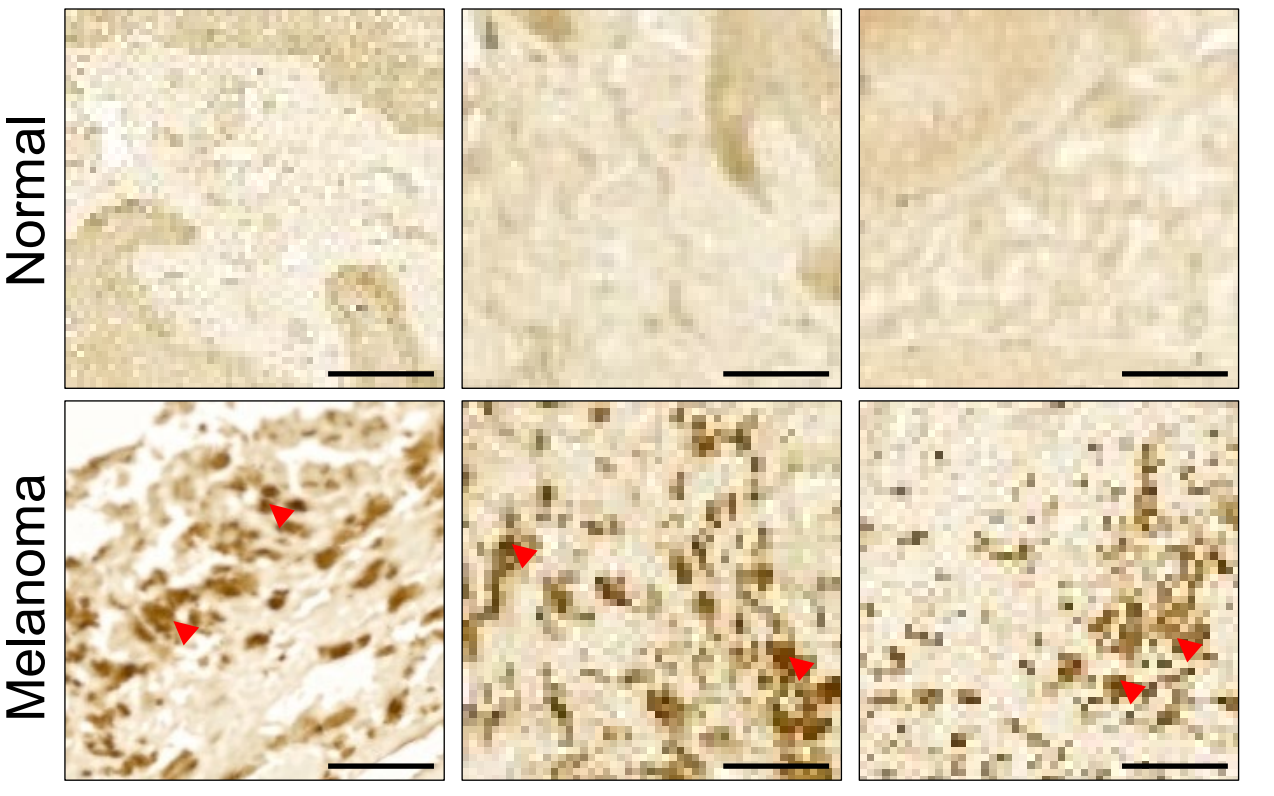

Revised Supplementary Figure 2

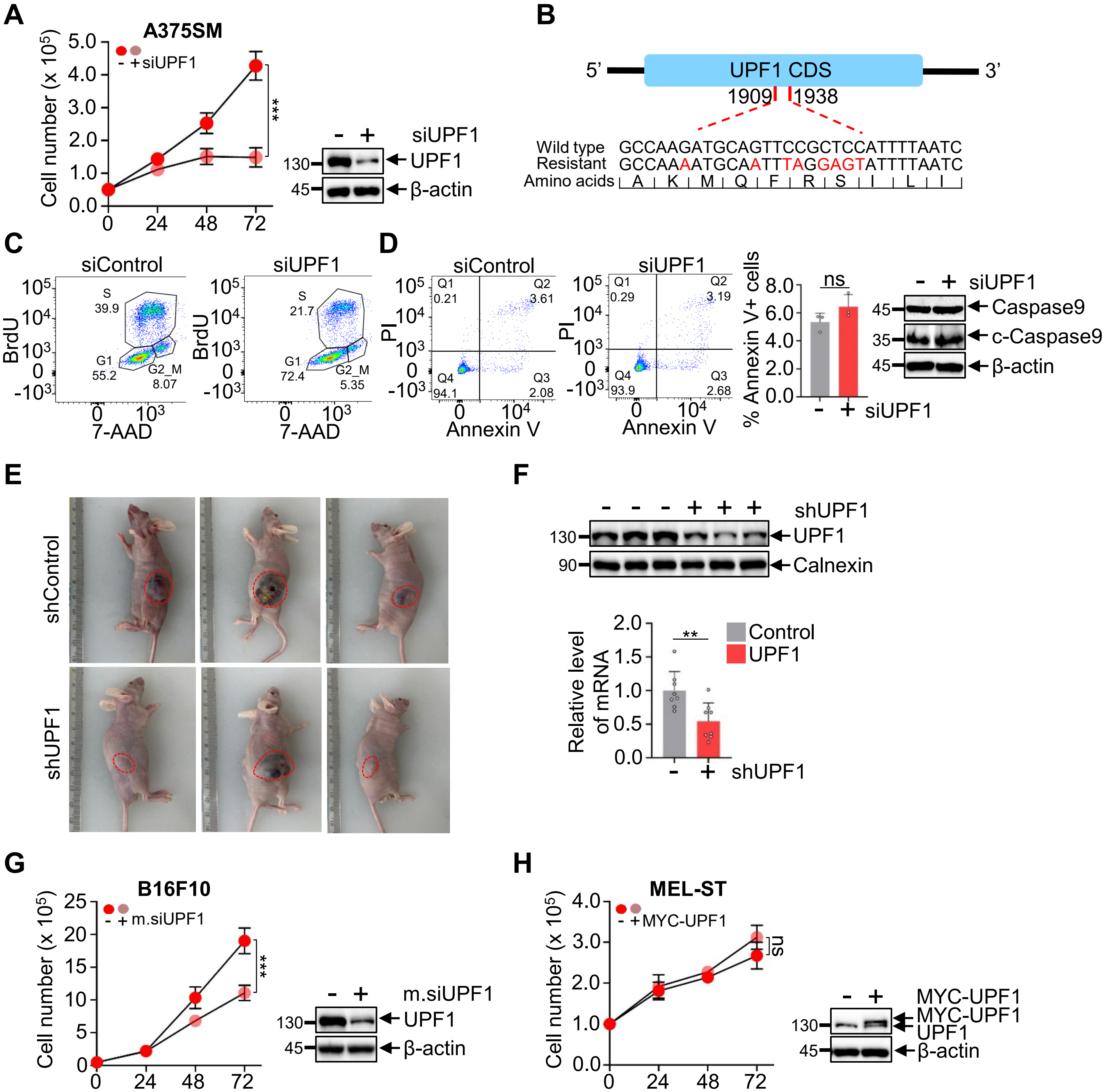

Revised Supplementary Figure 3

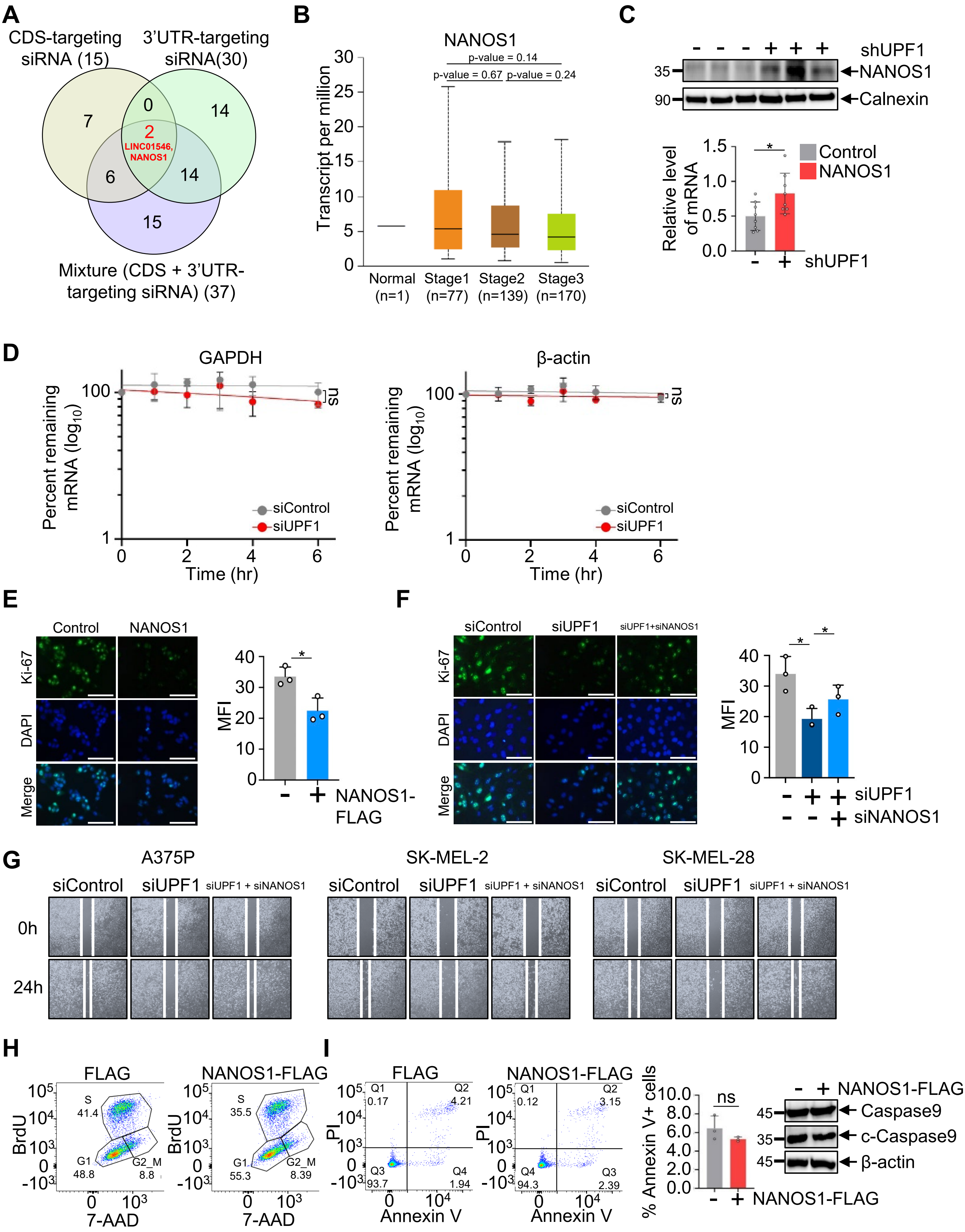

Revised Supplementary Figure 4

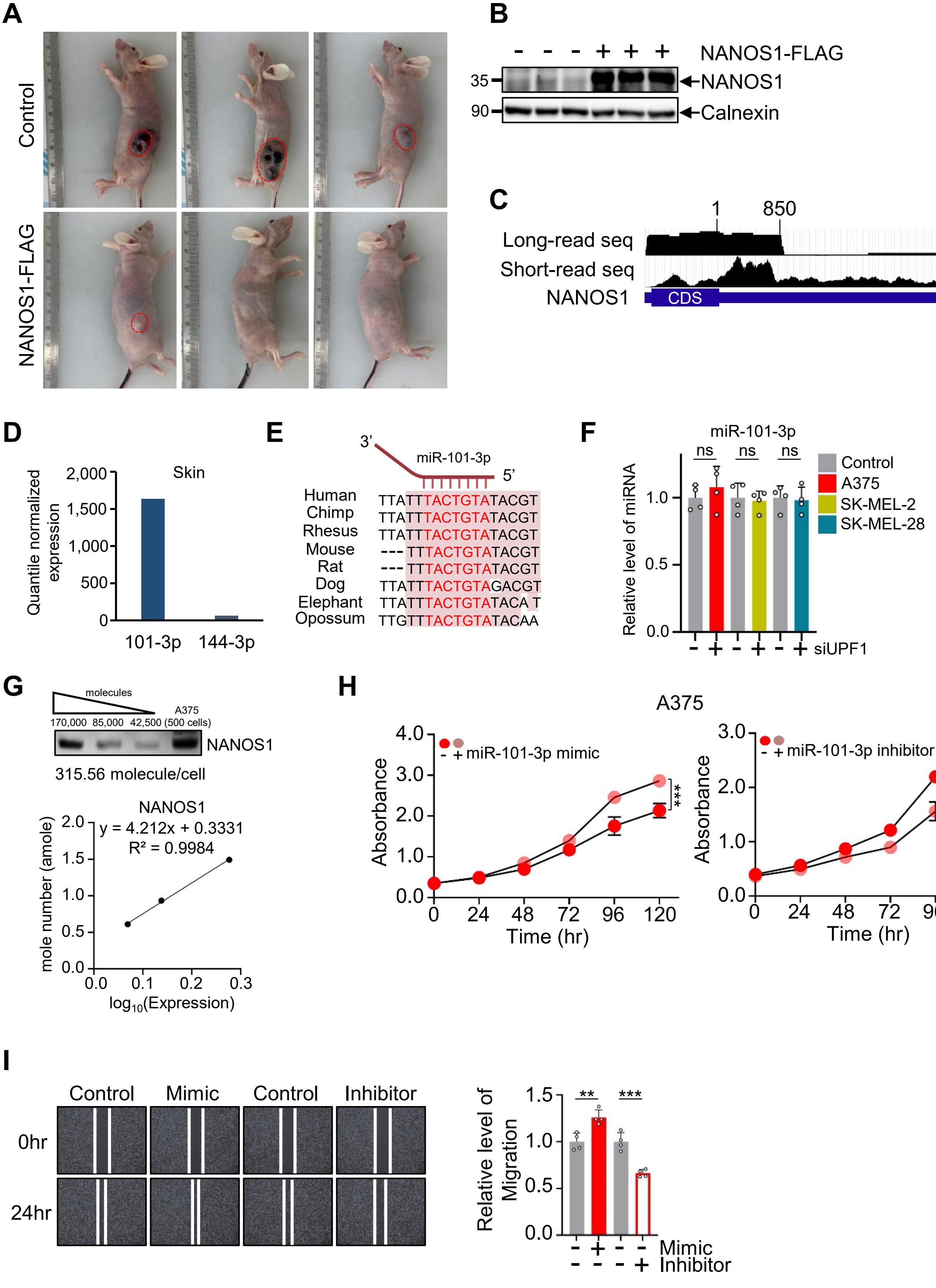

Revised Supplementary Figure 5

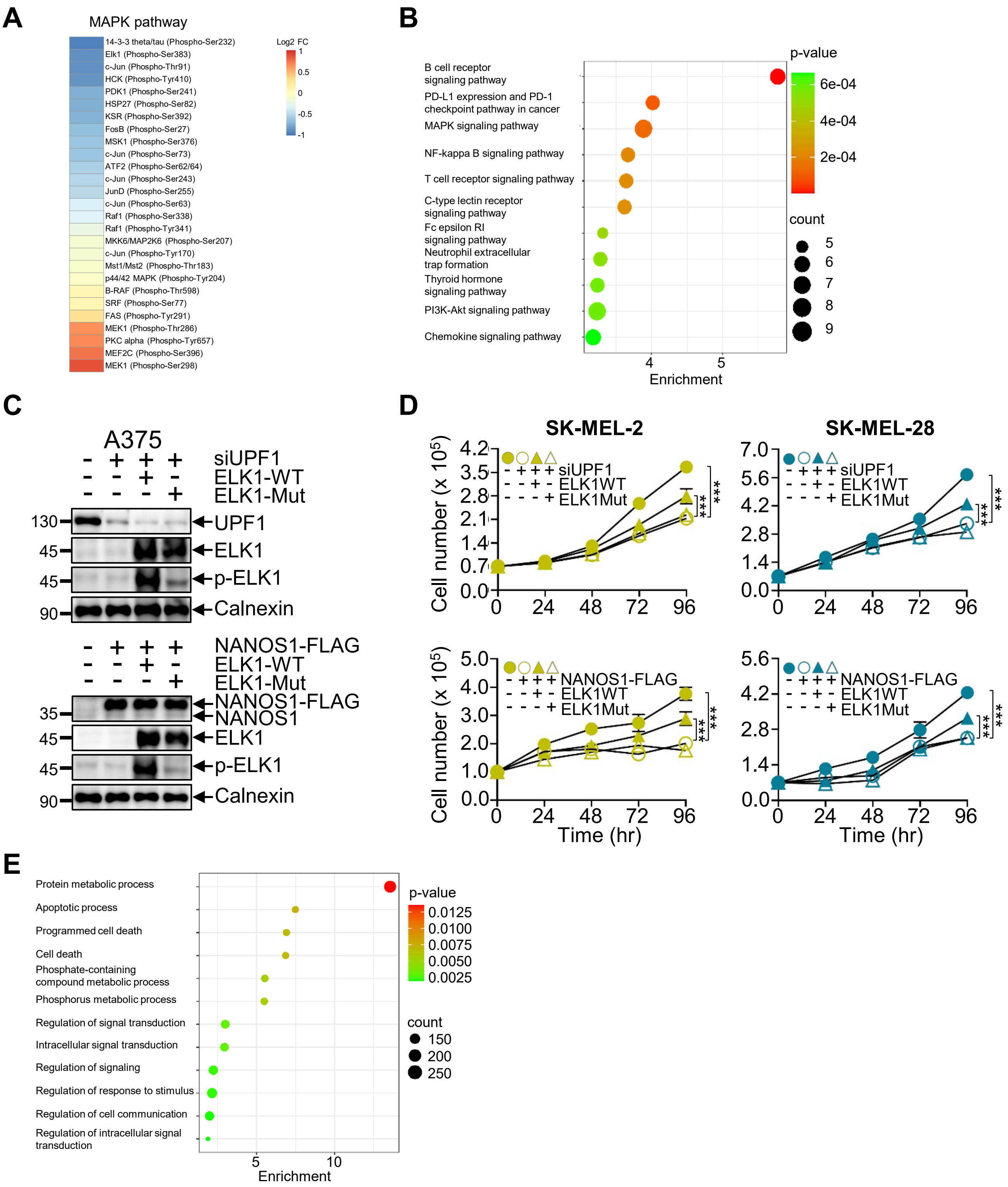

Revised Supplementary Figure 6

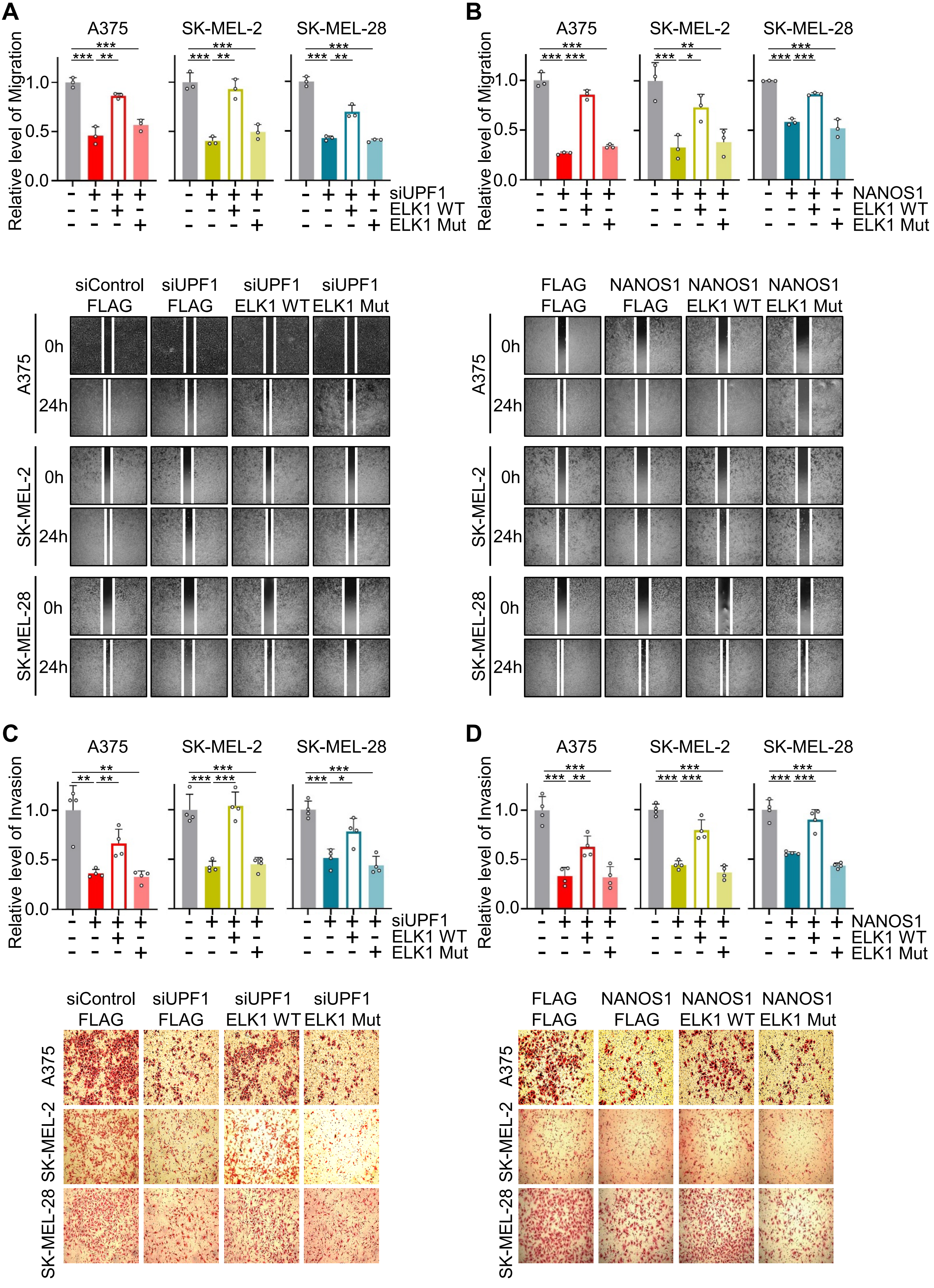

Revised Supplementary Figure 7

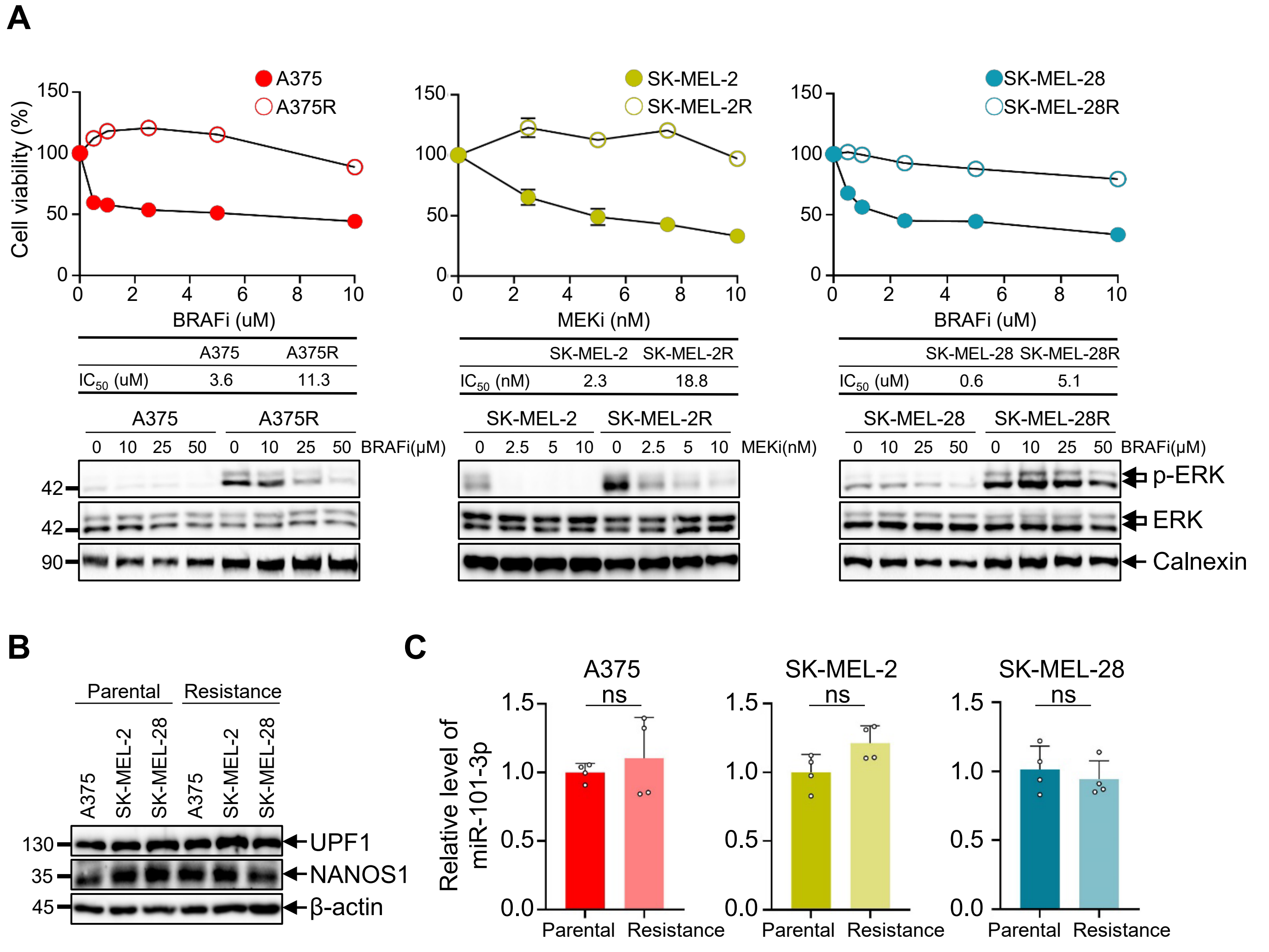

Revised Supplementary Figure 8

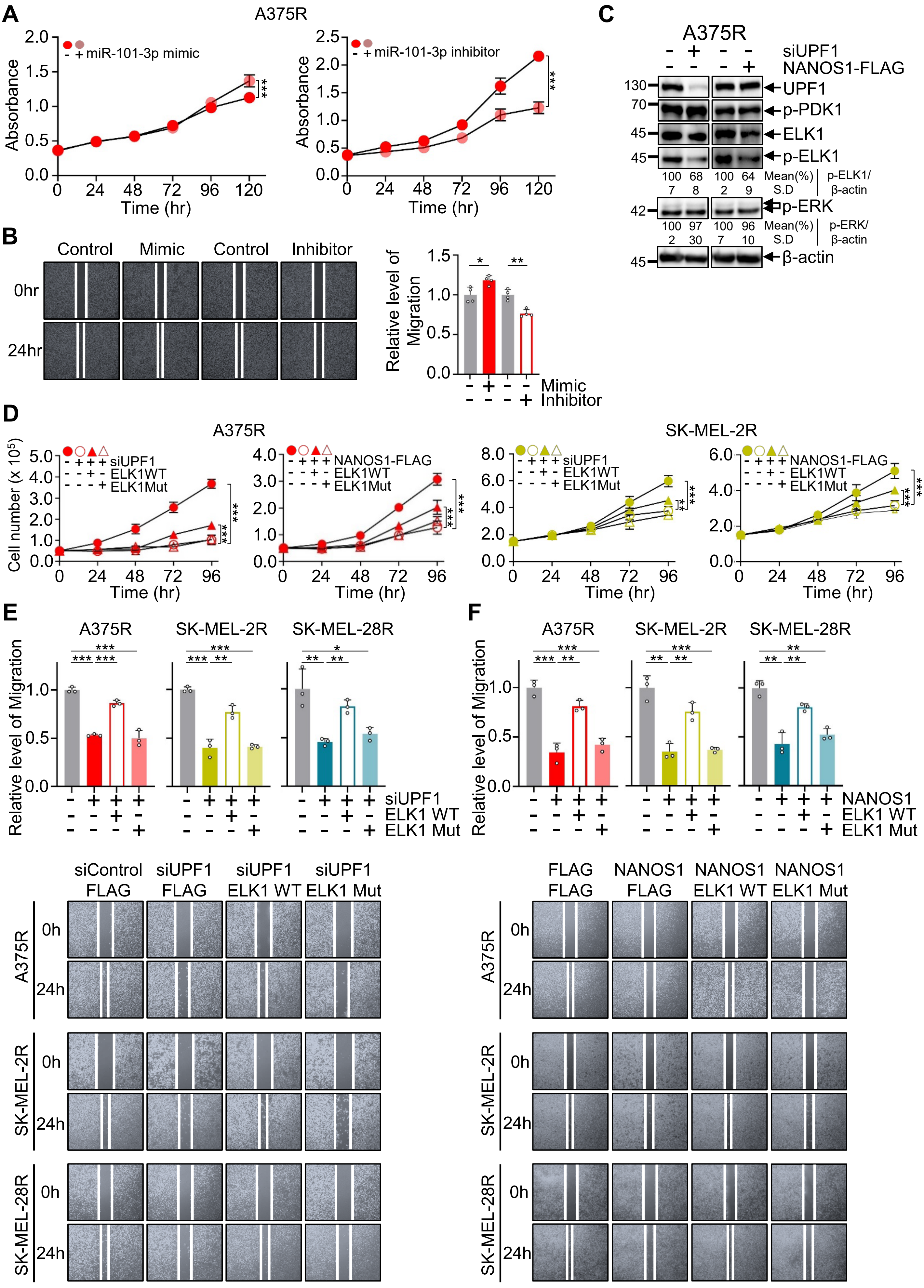

Table S1. RNA-seq analysis

Gene_Symbol	Log2F.C	p-value	FPKM			
			siControl-1	siControl-2	siUPF1_CDS-1	siUPF1_CDS-2
TRIM48	2.39	0.00	3.44	3.23	21.46	21.25
NANOS1	1.63	0.01	3.05	3.13	10.97	11.99
B3GNT7	1.30	0.04	30.62	25.86	74.93	67.66
ADAMTS1	1.29	0.04	3.06	2.56	8.27	8.12
ALDH1A3	1.18	0.05	168.58	197.44	429.83	425.98
CYP2E1	1.17	0.01	0.45	0.64	2.41	2.72
CDC42EP5	1.14	0.01	5.51	5.89	13.01	14.27
RPL23AP7	1.11	0.01	3.31	2.99	7.41	8.23
LINC01945	1.06	0.01	1.41	1.17	3.59	3.99
TRIML2	1.05	0.03	0.26	0.36	1.78	1.81
GABARAPL1	1.04	0.01	14.12	15.17	31.51	29.46
ZNF790-AS1	1.03	0.02	2.42	2.83	7.04	5.74
SUMO3	1.01	0.00	17.16	18.33	38.40	34.92
SPACA6	1.01	0.04	2.98	3.28	7.35	7.11
LINC01546	1.00	0.03	0.31	0.47	1.81	1.94
HGF	-1.01	0.03	35.88	32.22	16.46	16.41
ADAR	-1.01	0.00	91.26	96.30	46.30	46.83
STC1	-1.03	0.00	7.76	7.29	3.04	3.23
MYD88	-1.06	0.03	27.32	28.19	12.26	13.47
SPOCD1	-1.06	0.03	2.88	2.48	0.75	0.76
TSPAN13	-1.08	0.01	144.96	150.16	72.98	68.26
PI15	-1.08	0.01	29.60	29.17	13.76	13.11
TAC1	-1.10	0.02	21.64	17.74	9.28	7.87
IFI35	-1.12	0.03	95.43	103.87	46.71	45.90
APOL6	-1.14	0.00	4.82	4.70	1.65	1.58
G3BP2	-1.15	0.01	22.57	23.89	9.75	9.99
IFI16	-1.15	0.03	275.45	283.73	127.95	131.22
IL1RAP	-1.15	0.00	29.86	28.04	12.17	12.90
UBE2I	-1.17	0.00	24.43	25.53	10.75	10.19
C11orf98	-1.19	0.01	61.07	68.17	27.04	29.52
IFIT5	-1.20	0.00	10.18	11.00	4.20	3.85
B2M	-1.21	0.01	582.65	571.18	288.08	251.95
SPP1	-1.24	0.02	160.40	137.47	68.26	60.22
ISG20	-1.25	0.01	31.04	30.59	12.78	12.31
REC8	-1.26	0.01	3.62	3.44	0.88	0.89
NLRC5	-1.27	0.00	5.43	5.46	1.64	1.72
PHF11	-1.31	0.04	11.17	11.01	4.17	3.55
DCT	-1.32	0.03	73.45	61.41	25.80	27.96
ABHD17C	-1.34	0.01	51.40	56.59	21.66	19.87
DTX3L	-1.35	0.01	37.49	38.70	14.35	14.19

IFITM3	-1.35	0.01	1,051.63	1,106.47	473.82	469.97
BISPR	-1.39	0.02	5.07	5.31	1.22	1.53
TRIM34	-1.39	0.00	5.02	5.56	1.28	1.50
HLA-F	-1.44	0.02	31.40	26.85	9.77	10.42
GMPR	-1.44	0.03	3.15	3.16	0.45	0.59
NMI	-1.46	0.01	16.90	18.71	6.09	5.53
TAP1	-1.48	0.05	50.16	42.35	16.50	15.79
UBE2L6	-1.48	0.03	115.74	126.99	44.31	42.91
LAP3	-1.49	0.02	91.96	103.46	35.91	33.82
THEMIS2	-1.50	0.03	10.03	11.12	3.10	3.08
PNPT1	-1.51	0.00	43.96	42.79	14.98	14.30
WARS1	-1.53	0.01	242.35	249.07	85.94	86.03
PCSK1	-1.54	0.01	12.87	11.33	3.62	3.38
SP110	-1.56	0.01	30.43	31.88	9.55	10.37
PARP9	-1.57	0.03	50.11	54.12	17.40	16.84
TRIM14	-1.60	0.03	11.90	11.71	3.46	2.98
TRIM25	-1.61	0.00	38.92	40.24	12.25	12.40
GBP1	-1.61	0.01	12.39	12.73	3.61	3.23
HERC6	-1.63	0.05	5.23	6.30	1.18	1.19
LOC100419583	-1.64	0.01	18.50	20.03	5.99	5.01
STAT2	-1.65	0.02	63.67	64.69	19.48	20.62
BATF2	-1.80	0.02	5.27	5.41	0.72	0.91
PARP12	-1.83	0.00	26.67	28.78	6.96	7.24
SAMHD1	-1.84	0.00	17.70	18.49	4.47	4.22
TRANK1	-1.86	0.00	9.25	8.55	1.67	1.81
UPF1	-1.89	0.01	32.96	29.71	7.25	8.27
UBA7	-1.90	0.01	19.44	18.81	4.21	4.53
SAMD9	-1.93	0.00	8.69	9.16	1.52	1.71
EIF2AK2	-2.04	0.02	24.06	26.97	5.51	5.38
PARP14	-2.06	0.00	44.96	41.75	9.55	9.74
SAMD9L	-2.07	0.01	8.48	7.70	1.14	1.20
TRIM21	-2.11	0.01	18.86	20.15	3.84	3.68
TRIM22	-2.18	0.01	7.33	6.34	0.68	0.75
DHX58	-2.27	0.03	19.79	20.15	2.99	3.67
IRF9	-2.28	0.01	42.29	48.76	8.38	8.84
DDX60L	-2.29	0.01	17.14	16.62	2.55	2.81
PARP10	-2.30	0.01	54.85	51.58	10.16	10.00
HERC5	-2.52	0.00	17.20	17.64	2.21	2.27
RTP4	-2.54	0.01	13.58	15.35	1.94	1.41
PLSCR1	-2.57	0.00	86.12	93.47	14.26	14.97
IFIH1	-2.64	0.01	15.66	15.44	1.57	1.79
USP18	-2.86	0.02	24.24	23.97	2.73	2.29
STAT1	-2.90	0.00	163.91	173.70	22.99	21.99

IFIT2	-2.98	0.01	16.13	16.36	1.32	1.10
DDX60	-3.02	0.00	22.14	23.79	2.10	1.86
HELZ2	-3.17	0.00	59.66	56.11	5.43	5.91
MX1	-3.42	0.00	22.75	20.98	1.09	1.22
CMPK2	-3.58	0.01	14.70	15.97	0.36	0.36
BST2	-3.76	0.00	132.53	122.37	8.38	8.93
OAS3	-3.76	0.00	73.98	74.94	4.79	4.79
DDX58	-3.89	0.02	26.58	27.12	1.05	0.74
IFIT3	-4.19	0.00	67.99	71.26	2.85	3.18
IFIT1	-4.22	0.01	39.81	40.99	1.39	1.10
IFI44	-4.24	0.00	40.00	44.93	1.51	1.18
IRF7	-4.38	0.00	40.93	43.02	1.12	1.08
IFI6	-4.45	0.02	915.52	832.29	45.72	44.02
RSAD2	-4.45	0.02	24.41	19.72	0.03	0.06
OAS1	-4.52	0.00	105.90	110.24	3.96	3.86
XAF1	-4.55	0.01	29.29	26.34	0.23	0.23
IFITM1	-4.83	0.00	530.63	507.91	16.92	18.77
ISG15	-5.53	0.00	298.95	314.28	5.96	5.55
OAS2	-5.81	0.00	119.27	123.56	1.22	1.33
IFI27	-5.86	0.00	69.10	75.21	0.21	0.41

Gene_Symbol	Log2F.C	p-value	FPKM			
			siControl-1	siControl-2	siUPF1_3'UTR-1	siUPF1_3'UTR-2
RGS2	2.11	0.01	3.54	4.43	19.13	21.45
NANOS1	1.61	0.00	3.05	3.13	12.09	10.40
CHAC1	1.51	0.05	2.75	2.59	8.79	9.68
SYS1-DBNDD2	1.50	0.01	1.37	1.56	5.63	6.06
TGM2	1.49	0.01	3.89	4.09	13.03	12.28
RGS3	1.44	0.00	7.61	7.34	22.34	21.02
NGFR	1.40	0.01	6.54	5.53	18.69	15.84
CITED1	1.35	0.01	12.73	14.54	34.30	38.32
LRRC17	1.32	0.03	3.40	3.27	10.56	8.57
TP53I3	1.31	0.01	9.09	10.41	24.23	26.80
A2M	1.30	0.02	43.34	38.47	109.82	100.19
ADAM12	1.28	0.01	2.37	2.11	7.42	5.97
RGS4	1.27	0.00	0.06	0.09	1.73	1.44
SOCS2	1.26	0.00	7.29	7.48	19.68	17.89
ARHGDIG	1.21	0.03	0.96	1.19	3.81	3.58
CITED2	1.21	0.01	3.72	3.99	9.82	10.04
GADD45A	1.20	0.00	17.83	19.00	43.11	45.13
PINX1	1.18	0.01	9.96	9.80	24.38	22.86
JUN	1.18	0.04	3.48	3.50	8.75	9.09
RGS16	1.17	0.00	8.06	8.37	20.37	18.51
BAG1	1.16	0.01	4.68	5.08	11.55	12.17
CYP1A1	1.16	0.00	4.15	3.92	10.12	9.83
PDRG1	1.13	0.04	9.31	8.99	20.12	21.97
MRPL10	1.10	0.02	24.52	27.46	52.99	62.99
ATPAF1	1.09	0.03	11.41	11.53	24.56	26.22
PKIG	1.04	0.01	24.56	24.27	51.97	52.35
SBDSP1	1.03	0.02	9.35	8.26	17.94	19.72
CH17-340M24.3	1.03	0.04	2.54	3.24	6.20	7.21
STRA6	1.02	0.04	5.13	3.87	9.28	10.44
LINC01546	1.01	0.02	0.31	0.47	2.02	1.57
AIM2	-1.00	0.01	54.01	54.71	28.27	25.89
RRM2	-1.01	0.00	24.57	26.00	12.00	11.89
TRIM62	-1.01	0.04	5.82	5.08	2.23	1.97
SNORA32	-1.01	0.03	23.68	27.08	11.09	12.63
SAT1	-1.01	0.01	137.49	139.06	68.70	74.22
CD68	-1.02	0.04	132.81	113.26	64.45	62.04
MIR647	-1.02	0.03	7.14	5.80	2.81	2.24
SHISA5	-1.02	0.01	133.49	140.92	67.64	75.21
PLEKHA4	-1.02	0.00	39.04	37.90	18.55	18.10
POGLUT3	-1.03	0.04	8.91	7.15	3.96	2.63
RALB	-1.04	0.04	18.01	18.63	9.21	7.30

MASTL	-1.05	0.03	7.00	7.25	3.15	2.44
ST3GAL6	-1.06	0.01	25.46	24.11	11.83	10.84
TNFRSF21	-1.06	0.03	52.43	44.45	26.04	20.06
MRGPRX3	-1.08	0.03	30.21	24.70	11.92	12.79
SNORD5	-1.11	0.00	31.85	29.59	13.68	13.60
ORC6	-1.11	0.02	13.85	15.77	7.02	5.19
LHFPL3-AS1	-1.11	0.01	15.58	14.20	6.10	6.26
PIK3CB	-1.12	0.00	12.56	12.96	5.35	4.92
NCSTN	-1.13	0.03	117.50	95.54	54.20	45.83
CXCL1	-1.13	0.02	4.79	4.24	1.69	1.21
PMEL	-1.14	0.03	17.62	14.01	6.17	6.65
NLRC5	-1.14	0.00	5.43	5.46	1.89	1.73
CASP1	-1.17	0.02	2.16	2.62	0.44	0.54
NPPC	-1.17	0.02	24.53	21.26	9.51	9.33
MIR5047	-1.19	0.02	160.59	135.97	72.64	63.05
BATF2	-1.19	0.04	5.27	5.41	1.58	1.75
CEMIP2	-1.20	0.05	45.20	35.57	20.03	13.87
TMBIM6	-1.21	0.04	243.60	205.48	105.08	103.63
TLR3	-1.23	0.03	4.70	3.58	1.36	0.92
SERINC5	-1.24	0.04	16.57	12.83	6.30	4.70
SNORD87	-1.26	0.02	24.20	24.66	9.37	9.57
MIR3142HG	-1.29	0.01	67.99	63.70	28.37	26.37
HLA-F	-1.28	0.05	31.40	26.85	10.08	12.47
GRAMD1B	-1.31	0.02	59.18	52.03	23.28	21.19
WARS1	-1.32	0.04	242.35	249.07	108.13	101.10
IFI35	-1.34	0.01	95.43	103.87	37.51	42.61
STAT2	-1.35	0.04	63.67	64.69	27.56	23.19
SP100	-1.36	0.02	51.11	42.47	19.54	15.89
THSD4	-1.36	0.04	5.06	3.90	1.19	0.94
ERP44	-1.42	0.01	14.20	11.94	4.08	4.05
UBE2L6	-1.42	0.02	115.74	126.99	43.16	50.48
THEMIS2	-1.43	0.02	10.03	11.12	2.92	3.32
TM4SF1	-1.46	0.03	122.54	99.49	43.75	38.69
PNPT1	-1.46	0.00	43.96	42.79	15.42	14.81
LAP3	-1.47	0.01	91.96	103.46	37.52	34.32
ABHD17C	-1.51	0.03	51.40	56.59	18.93	17.97
SP110	-1.55	0.00	30.43	31.88	10.88	9.10
GMPR	-1.56	0.01	3.15	3.16	0.35	0.42
TREX1	-1.59	0.02	3.39	4.31	0.44	0.69
GBP1	-1.61	0.01	12.39	12.73	3.46	3.15
REC8	-1.62	0.02	3.62	3.44	0.38	0.51
BISPR	-1.62	0.03	5.07	5.31	1.16	0.78
HERC6	-1.63	0.04	5.23	6.30	1.19	1.02

TRIM25	-1.63	0.02	38.92	40.24	13.17	10.70
TRIM34	-1.64	0.01	5.02	5.56	0.85	1.03
PARP12	-1.66	0.00	26.67	28.78	7.95	7.96
UPF1	-1.68	0.01	32.96	29.71	8.84	9.20
LOC100419583	-1.68	0.00	18.50	20.03	5.21	5.06
SAMHD1	-1.74	0.01	17.70	18.49	4.80	4.31
PCSK1	-1.86	0.00	12.87	11.33	2.84	2.16
SAMD9	-1.86	0.00	8.69	9.16	1.79	1.52
TRIM14	-1.90	0.01	11.90	11.71	2.51	2.12
TRIM22	-1.91	0.00	7.33	6.34	0.95	1.08
TRANK1	-1.94	0.00	9.25	8.55	1.70	1.33
TRIM21	-2.05	0.00	18.86	20.15	3.75	3.82
EIF2AK2	-2.07	0.03	24.06	26.97	6.34	4.04
IRF9	-2.09	0.02	42.29	48.76	8.83	10.91
PARP9	-2.11	0.01	50.11	54.12	12.77	9.93
PARP14	-2.15	0.00	44.96	41.75	9.44	8.22
CEMIP	-2.19	0.01	270.10	212.43	61.35	48.61
DHX58	-2.25	0.00	19.79	20.15	3.32	3.23
RTP4	-2.25	0.00	13.58	15.35	2.11	2.18
PLSCR1	-2.27	0.00	86.12	93.47	19.02	17.13
PARP10	-2.30	0.00	54.85	51.58	10.09	9.85
DDX60L	-2.40	0.02	17.14	16.62	2.63	1.97
IFIH1	-2.44	0.02	15.66	15.44	2.21	1.73
USP18	-2.46	0.00	24.24	23.97	3.63	3.25
HERC5	-2.57	0.00	17.20	17.64	2.11	1.92
SAMD9L	-2.57	0.00	8.48	7.70	0.61	0.40
IFIT2	-2.74	0.03	16.13	16.36	1.83	1.25
STAT1	-2.88	0.01	163.91	173.70	23.15	22.47
DDX60	-3.00	0.00	22.14	23.79	2.12	1.74
CMPK2	-3.25	0.00	14.70	15.97	0.64	0.73
MX1	-3.33	0.01	22.75	20.98	1.27	1.15
BST2	-3.65	0.00	132.53	122.37	9.44	9.08
HELZ2	-3.68	0.00	59.66	56.11	3.89	3.25
IFIT3	-3.96	0.00	67.99	71.26	3.88	3.27
DDX58	-4.00	0.00	26.58	27.12	0.75	0.71
OAS3	-4.08	0.00	73.98	74.94	3.66	3.34
IFI44	-4.15	0.00	40.00	44.93	1.31	1.47
IRF7	-4.15	0.01	40.93	43.02	1.26	1.47
IFIT1	-4.32	0.00	39.81	40.99	1.12	0.96
OAS1	-4.36	0.02	105.90	110.24	4.04	4.52
RSAD2	-4.46	0.02	24.41	19.72	0.05	0.03
IFITM1	-4.47	0.00	530.63	507.91	20.74	25.84
XAF1	-4.53	0.01	29.29	26.34	0.26	0.24

IFI6	-5.06	0.01	915.52	832.29	29.14	29.66
ISG15	-5.59	0.04	298.95	314.28	3.80	6.79
OAS2	-5.68	0.00	119.27	123.56	1.53	1.26
IFI27	-5.97	0.00	69.10	75.21	0.18	0.24

Gene_Symbol	Log2F.C	p-value	FPKM			
			siControl-1	siControl-2	siUPF1_Mixture-1	siUPF1_Mixture-2
TRIM48	1.83	0.00	3.44	3.23	13.88	13.97
NANOS1	1.72	0.00	3.05	3.13	12.29	11.72
SOCS2-AS1	1.68	0.04	1.73	2.51	9.78	7.93
RGS2	1.59	0.02	3.54	4.43	14.92	12.26
LRRC17	1.55	0.01	3.40	3.27	11.27	11.26
MEF2C	1.40	0.05	5.23	6.44	16.69	16.24
RGS3	1.32	0.03	7.61	7.34	21.32	17.63
ADAM12	1.30	0.02	2.37	2.11	7.02	6.65
B3GNT7	1.30	0.04	30.62	25.86	74.54	65.15
SYS1-DBNDD2	1.28	0.01	1.37	1.56	4.78	5.10
RPL23AP7	1.28	0.02	3.31	2.99	9.07	8.60
A2M	1.27	0.03	43.34	38.47	100.33	94.10
OPN3	1.25	0.00	13.48	13.78	33.86	31.55
TRIML2	1.24	0.02	0.26	0.36	2.20	2.03
CHAC1	1.19	0.01	2.75	2.59	7.65	6.68
PINX1	1.19	0.00	9.96	9.80	23.94	21.99
TRNV	1.16	0.04	34.17	43.62	80.85	90.90
RRM2B	1.15	0.04	9.82	11.57	24.32	23.92
BAG1	1.14	0.00	4.68	5.08	12.49	10.86
TRNF	1.14	0.00	23.74	23.97	53.76	50.81
ZNF790-AS1	1.14	0.05	2.42	2.83	7.98	5.78
ZFAND2A	1.11	0.03	6.94	6.98	16.97	14.42
LINC01546	1.11	0.03	0.31	0.47	1.96	2.05
MGP	1.11	0.00	90.75	87.69	205.63	181.16
HEIH	1.07	0.03	6.65	8.22	18.19	14.28
THEM4	1.07	0.00	16.85	16.82	37.41	33.50
RGS16	1.07	0.00	8.06	8.37	18.62	16.76
CITED1	1.06	0.02	12.73	14.54	31.70	25.61
TENT5C	1.06	0.01	0.86	0.96	3.24	2.71
SOCS2	1.05	0.00	7.29	7.48	16.58	15.06
TGM2	1.04	0.01	3.89	4.09	9.76	8.35
CITED2	1.04	0.02	3.72	3.99	8.92	8.55
ISY1-RAB43	1.03	0.03	0.49	0.49	2.22	1.91
RHOB	1.02	0.00	10.75	11.26	24.22	21.24
SUMO3	1.01	0.03	17.16	18.33	37.29	34.02
SSUH2	1.01	0.01	6.02	5.46	11.99	12.17
ADAMTS1	1.00	0.05	3.06	2.56	6.66	6.22
PTGES	-1.00	0.03	30.64	27.10	14.51	12.93
TNFAIP3	-1.01	0.00	10.67	10.10	4.74	4.34
B2M	-1.02	0.00	582.65	571.18	295.18	283.95
CHROMR	-1.02	0.02	9.93	10.27	4.46	4.25

CCN3	-1.02	0.01	11.86	11.18	5.41	4.70
GIMAP2	-1.03	0.05	2.94	3.87	0.90	1.34
SECTM1	-1.04	0.04	7.96	7.03	3.07	3.02
UBE2I	-1.05	0.01	24.43	25.53	12.27	10.48
AIM2	-1.06	0.00	54.01	54.71	26.23	24.08
PRDM7	-1.07	0.04	15.12	12.30	5.73	5.97
CXCL1	-1.08	0.01	4.79	4.24	1.43	1.63
ADAR	-1.09	0.01	91.26	96.30	44.57	41.60
IFITM3	-1.09	0.01	1,051.63	1,106.47	557.32	518.33
SLC30A1	-1.09	0.02	22.76	20.38	9.46	9.28
NMI	-1.11	0.04	16.90	18.71	7.86	7.27
IGFBP3	-1.13	0.01	67.47	60.83	28.65	29.12
SNORD49A	-1.13	0.00	26.28	26.03	11.45	10.92
IMPAD1	-1.15	0.01	30.93	27.48	12.21	12.45
NLRC5	-1.16	0.02	5.43	5.46	1.92	1.70
IL1RAP	-1.16	0.01	29.86	28.04	11.94	12.40
IFI35	-1.18	0.01	95.43	103.87	47.88	39.25
ISG20	-1.19	0.02	31.04	30.59	13.61	11.98
UGCG	-1.21	0.05	18.89	16.13	6.95	6.69
LOC100310756	-1.25	0.02	11.67	12.29	4.09	4.58
MIR3142HG	-1.27	0.03	67.99	63.70	25.38	27.87
WARS1	-1.28	0.00	242.35	249.07	106.73	95.44
STAT2	-1.29	0.01	63.67	64.69	26.07	25.33
ERP44	-1.32	0.03	14.20	11.94	4.48	4.52
PMEL	-1.32	0.02	17.62	14.01	6.41	4.80
TAP1	-1.33	0.04	50.16	42.35	18.67	16.49
TAC1	-1.36	0.04	21.64	17.74	6.81	6.91
TM4SF1	-1.37	0.03	122.54	99.49	41.07	43.23
DTX3L	-1.37	0.00	37.49	38.70	14.00	13.45
BISPR	-1.38	0.00	5.07	5.31	1.38	1.28
UBE2L6	-1.38	0.02	115.74	126.99	47.57	44.22
TRIM34	-1.43	0.01	5.02	5.56	1.17	1.39
REC8	-1.43	0.00	3.62	3.44	0.68	0.62
LAP3	-1.43	0.02	91.96	103.46	37.70	33.22
DCT	-1.45	0.04	73.45	61.41	24.14	23.88
SP110	-1.48	0.00	30.43	31.88	10.69	10.31
BATF2	-1.50	0.01	5.27	5.41	1.30	1.09
PNPT1	-1.51	0.02	43.96	42.79	14.10	14.54
ABHD17C	-1.55	0.03	51.40	56.59	18.08	17.01
TRIM25	-1.56	0.00	38.92	40.24	12.78	12.14
LOC100419583	-1.56	0.02	18.50	20.03	5.90	5.60
GBP1	-1.58	0.04	12.39	12.73	3.90	3.01
HLA-F	-1.59	0.04	31.40	26.85	9.24	8.53

THEMIS2	-1.60	0.01	10.03	11.12	2.89	2.63
HERC6	-1.63	0.05	5.23	6.30	1.15	1.11
GMPR	-1.63	0.02	3.15	3.16	0.37	0.27
PARP12	-1.68	0.00	26.67	28.78	8.50	7.30
SAMD9	-1.71	0.01	8.69	9.16	1.97	1.95
PCSK1	-1.75	0.03	12.87	11.33	2.89	2.76
TRANK1	-1.77	0.01	9.25	8.55	1.89	1.78
TRIM14	-1.77	0.00	11.90	11.71	2.71	2.65
PARP9	-1.78	0.00	50.11	54.12	14.43	14.25
UBA7	-1.82	0.00	19.44	18.81	4.60	4.60
TREX1	-1.83	0.01	3.39	4.31	0.43	0.25
SAMHD1	-1.83	0.01	17.70	18.49	4.64	3.93
EIF2AK2	-1.96	0.03	24.06	26.97	5.80	5.56
UPF1	-2.01	0.01	32.96	29.71	7.31	6.64
IRF9	-2.06	0.01	42.29	48.76	9.50	10.65
TRIM22	-2.09	0.01	7.33	6.34	0.74	0.84
PARP14	-2.09	0.01	44.96	41.75	8.91	9.63
DDX60L	-2.16	0.00	17.14	16.62	3.00	2.88
TRIM21	-2.20	0.01	18.86	20.15	3.76	3.07
CEMIP	-2.23	0.05	270.10	212.43	53.80	48.58
SAMD9L	-2.29	0.00	8.48	7.70	0.78	0.85
PARP10	-2.33	0.00	54.85	51.58	9.97	9.55
PLSCR1	-2.35	0.01	86.12	93.47	17.28	16.31
DHX58	-2.38	0.00	19.79	20.15	3.06	2.84
RTP4	-2.41	0.00	13.58	15.35	1.73	1.97
IFIH1	-2.42	0.02	15.66	15.44	1.91	2.16
USP18	-2.48	0.00	24.24	23.97	3.58	3.33
HERC5	-2.49	0.00	17.20	17.64	2.29	2.17
IFIT2	-2.65	0.01	16.13	16.36	1.80	1.59
STAT1	-2.87	0.01	163.91	173.70	23.41	21.62
DDX60	-2.96	0.00	22.14	23.79	2.17	1.90
CMPK2	-3.26	0.00	14.70	15.97	0.61	0.77
MX1	-3.39	0.01	22.75	20.98	1.17	1.14
HELZ2	-3.59	0.00	59.66	56.11	4.10	3.74
BST2	-3.80	0.00	132.53	122.37	8.89	7.58
DDX58	-3.82	0.00	26.58	27.12	0.94	0.98
IFIT3	-4.00	0.00	67.99	71.26	3.76	3.25
OAS3	-4.09	0.00	73.98	74.94	3.80	3.33
IFI44	-4.21	0.00	40.00	44.93	1.26	1.41
IFIT1	-4.25	0.00	39.81	40.99	1.13	1.19
IRF7	-4.39	0.00	40.93	43.02	0.96	1.13
RSAD2	-4.44	0.02	24.41	19.72	0.07	0.05
OAS1	-4.44	0.00	105.90	110.24	3.95	4.25

IFITM1	-4.51	0.00	530.63	507.91	21.16	22.69
XAF1	-4.58	0.00	29.29	26.34	0.23	0.18
IFI6	-4.73	0.01	915.52	832.29	37.90	33.79
ISG15	-5.62	0.00	298.95	314.28	5.55	5.00
OAS2	-5.62	0.01	119.27	123.56	1.68	1.35
IFI27	-5.84	0.00	69.10	75.21	0.28	0.36

Table S2. siRNA used in this study

siRNA name	Sense (5’ to 3’)
UPF1 CDS	CCAAGAUGCAGUUCCGCUCCAUU
UPF1 3’UTR	GCUUAGUCCAUCAGCAUCUUAUUCU
NANOS1 CDS-1	AACGCGCACACCAUCAAGUAC
NANOS1 CDS-2	GCUCUACACCACCCAUAUCCU
SMG7 CDS	GCAAGAAACAUCUGUGAUA

Table S3. Cloning primers used in this study

Primer name	Sequence (5’ to 3’)
EGFP-F	GCG<u>AAGCTT</u>ATGAGCAAGGGCGAGGAG
EGFP-R	GCG<u>GGATCC</u>TTACTTGTACAGCTCGTCC
NANOS1-3UTR-F	GCG<u>GGATCC</u>AGGCCCGGGCTCCCGGCC
NANOS1-3UTR-1-R	GCG<u>CTCGAG</u>TTACAGGTGAGATTAATGAATTCAT
NANOS1-3UTR-2-R	GCG<u>CTCGAG</u>AGAGGTAAGAGGATTCATGTTGAC
NANOS1-3UTR-3-R	GCG<u>CTCGAG</u>TATTTGTGATATAATTTTAATATGT
NANOS1-3UTR-miRNA-F	GCTCGCTAGCCTCGAGTGTATTCCACTAACTGAAATATGGC
NANOS1-3UTR-miRNA-R	CGACTCTAGACTCGAGATTGAGACAATTTCATACTGATGCG
ELK1-WT-F	CGATGACGACAAGCTTGACCCATCTGTGACGCTGTGG
ELK1-WT-R	ATGCCACCCGGGATCCTCATGGCTTCTGGGGCCC
ELK1-Mut-F	CTGGAGCACCCTGGCTCCCATTGCGCCC
ELK1-Mut-R	GGGCGCAATGGGAGCCAGGGTGCTCCAG
Hygro-F	CAGTGTGGTGGAATTCATGGCCAAAAAGCCTGAACTCAC
Hygro-R	GAGGTTGATTGTCGACCGGTCCAGGATTCTCTTCGACATC
NANOS1-Lenti-F	GAGAATCCTGGACCGATGGAGGCTTTCCCCTGGG
NANOS1-Lenti-R	GAGGTTGATTGTCGACTTACTTGTCGTCATCGTCTTTGTAG
NANOS1-Retro-F	CGGTGACTAGCTCGAATGGAGGCTTTCCCCTGG
NANOS1-Retro-R	CAAGCTTGAGCTCGATTACTTGTCGTCATCGTCTTTGTAG

Table S4. Oligo used in this study

Primer name	Sequence (5’ to 3’)
BamHI-FLAG-EcoRI	GCGGGATCCGACTACAAAGACGATGACGACAAGTAAGAATTCGCG
NANOS1-3UTR-WT	ATTGAGACAATTTCATACTGATGCGCATCTAAAATAGGTCGACGTATACAGTAAATAAACAGCGCCTCTAAGTTGCCATATTTCAGTTAGTGGAATACA
NANOS1-3UTR-Mut	ATTGAGACAATTTCATACTGATGCGCATCTAAAATAGGTCGACGTAATGTCATAATAAACAGCGCCTCTAAGTTGCCATATTTCAGTTAGTGGAATACA
h.shUPF1	TCGAGAAGGTATATTGCTGTTGACAGTGAGCGCTTAGTCCATCAGCATCTTATTTAGTGAAGCCACAGATGTAAATAAGATGCTGATGGACTAAGTGCCTACTGCCTCGG
m.shUPF1	TCGAGAAGGTATATTGCTGTTGACAGTGAGCGAAGCAGCTAATCCTCGTCGGTGTAGTGAAGCCACAGATGTACACCGACGAGGATTAGCTGCTTTGCCTACTGCCTCGG

Table S5. qPCR primers used in this study

qPCR primer name	Forward (5' to 3')	Reverse (3' to 5')
UPF1	GAGGCCGACTACGACAAGAA	CATGAGCCGCATGTCAGAGT
NANOS1	GAACAACAAGGAGGCGATGG	CTTTGGAGAGCGGGCAGTAC
SMG7	GGCAGGCAGAAGTCCTGAAG	AGGCGTGATTCCAGAGATCC
HDAC9	GTGGCCAAAGAGTTTGATCC	TGACCAAAACATTTTGCCGT
EGFP	ACCCTCGTGACCACCCTGACCT	GGCGCGGGTCTTGTAGTTGCCG
Firefly	CATGGATAGCAAGACCGACTAC	TCAGGGCGATGGTTTTGTC
Renilla	AGATCATGCGGAAACTGGAG	CGAAGGTAGGCGTTGTAGTTG
miR-101-3p	GCCGCCTACAGTACTGTG	GTGCAGGGTCCGAGGT
GAPDH	CAAGATCATCAGCAATGCC	CTGTGGTCATGAGTCCTTCC
MAP2K6	GGCTACTTGGTGGACTCTGTTG	CATCGTGATGCCCAGACTCCAA
NLK	CACTGGAAGCAATGAGGACAGC	ACAGCTTCATGTGTAGCCTGGC
FOSL2	AAGAGGAGGAGAAGCGTCGCAT	GCTCAGCAATCTCCTTCTGCAG
MAPK1	ACACCAACCTCTCGTACATCGG	TGGCAGTAGGTCTGGTGCTCAA
PPP3R1	CTGCCTGAGTTACAACAGAATCC	TGACACTGAACTGAGAGACGCC
CDC42	TGTTGTTGTGGGCGATGGTG	CTCCACCAATCATAACTGTGACTGC
NRAS	GAAACCTCAGCCAAGACCAGAC	GGCAATCCCATACAACCCTGAG
RAP1A	ACTTACAGGACCTGAGGGAACAG	CCTGCTCTTTGCCAACTACTCG
CASP3	GGAAGCGAATCAATGGACTCTGG	GCATCGACATCTGTACCAGACC
LAMTOR3	GTGGTGAGTTTCATAGCCAGCAG	AGAAACTTCCACAACTTGTCTCAG

Table S6. RT primers used in this study

Primer name	Sequence (5' to 3')
miR-101-3p stem-loop RT primer	GTCGTATCCAGTGCAGGGTCCGAGGTATTCGCACTGGATACGAC TTCAGT
